# Electrochemical imaging of thermochemical catalysis

**DOI:** 10.1038/s41929-026-01486-y

**Published:** 2026-03-05

**Authors:** Xiangdong Xu, William C. Howland, Daniel Martín-Yerga, Cole S. Cadaram, Deiaa M. Harraz, Geoff D. West, Patrick R. Unwin, Yogesh Surendranath

**Affiliations:** 1 Department of Chemistry, University of Warwick, Coventry, UK; 2 Department of Chemistry, Massachusetts Institute of Technology, Cambridge, MA USA; 3 Department of Chemistry and Materials Science, School of Chemical Engineering, Aalto University, Espoo, Finland; 4 Warwick Manufacturing Group, University of Warwick, Coventry, UK

**Keywords:** Electrocatalysis, Imaging studies, Electrocatalysis, Heterogeneous catalysis, Fuel cells

## Abstract

Thermochemical redox catalysis is critical to a wide array of key chemical transformations and can proceed via the coupling of two electrochemical half-reactions. This electrochemical mechanism is exemplified by the platinum-catalysed aerobic oxidation of formic acid, wherein the oxygen reduction reaction is coupled to the formic acid oxidation reaction. Here using scanning electrochemical cell microscopy, we show there are grain-dependent variations in catalytic rates for the oxygen reduction and formic acid oxidation reactions at a platinum catalyst. Quantitative spatially resolved images of catalytic rates imply inter-grain cooperativity during ensemble thermochemical catalysis via lateral current flows that galvanically couple disparate active sites. Moreover, by comparing current–potential profiles of the half-reactions in isolation and in the presence of both reactants, we reveal additional site-specific chemical interactions that modify the two constituent half-reactions. These studies establish a methodology that exposes how electrochemical half-reactions couple and interact across surface structures to enable redox transformations.

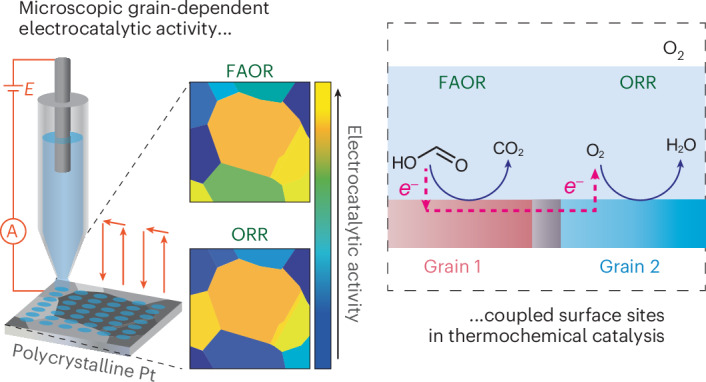

## Abstract (web summary)

Several thermochemical redox reactions have been shown to operate via the coupling of electrochemical half-reactions. Here, scanning electrochemical cell microscopy is employed to image the variations in catalytic rate at different grains of polycrystalline platinum during aerobic formic acid oxidation.

## Main

Thermochemical redox catalysis is critical across the chemical value chain with applications ranging from the generation of small molecule energy carriers to the synthesis of value-added fine chemicals. In particular, redox catalysis underpins modern synthetic methods for functional group interconversion and cross-couplings that simultaneously increase reaction scopes and improve atom economy^1–6^. In addition, hydrogenation and oxidation reactions of key energy-relevant molecules, including N_2_, CO_2_, O_2_, lignin and sugars are essential for the sustainable synthesis of fuels and commodity chemicals^7–13^. Metal surfaces are excellent catalysts for a wide variety of these thermochemical redox reactions. However, our understanding of heterogeneous redox catalysis remains limited, in part, because of the heterogeneity of active sites on display at most metal surfaces. Thus, strategies for mapping catalytic activity for these reactions across a surface would advance fundamental understanding, and potentially enable the rational design of tailored heterogeneous redox catalysts with enhanced performance.

Extensive investigations have focused on achieving spatially resolved operando measurements of heterogeneous catalytic systems. A variety of techniques have been developed depending on the length scale of interest^14^. Magnetic resonance imaging methods provide insight into the impact of mass transfer phenomena on product accumulation and inhomogeneity of reaction selectivities within millimetre-scale reactors with spatial resolution of a few hundred micrometres^15,16^. Observation of inhomogeneities on a nanometre to micrometre length scale, necessary for discrimination of active sites and chemical microenvironments, has generally been pursued with various forms of microscopy. Electron microscopy techniques such as high-resolution transmission electron microscopy and photoemission electron microscopy can report on the state of the catalyst surface on these length scales^17^. High-resolution transmission electron microscopy provides the capability to observe changes to catalyst morphology under realistic reaction conditions^17,18^. Photoemission electron microscopy provides information about surface speciation, but is only practical under near-vacuum conditions^17,19^. While these electron microscopy techniques provide invaluable information about catalyst structure and adsorbate populations, they provide little direct spatially resolved insight into local catalytic rates. Instead, strategies for resolving spatial inhomogeneities in thermocatalytic reaction rate have relied primarily on confocal optical microscopies (infrared, ultraviolet-visible light (UV-vis) and fluorescence)^14,20–23^. These techniques, which image spectroscopic signatures of products or on-cycle reaction intermediates, provide rich information about rate heterogeneities, particularly for photocatalytic reactions^20,24^. However, these techniques are restricted to those reactions for which kinetically relevant spectroscopic signatures exist natively or can be introduced with minimal perturbation. In addition, the diffusion of spectroscopic reporters on the catalyst surface or in solution must be taken into account in this analysis^20^. The enormous insights offered by these spatially resolved catalytic microscopies motivate the development of new imaging methodologies that leverage orthogonal and complementary modes for quantifying local catalytic rates.

Recent work has shown that a diverse collection of heterogeneously catalysed thermochemical redox reactions proceed by electrochemical band-mediated half-reaction mechanisms^25–31^. In such mechanisms, the underlying electrochemical half-reactions may be independently investigated as electrolytic processes using voltammetric techniques. The growing understanding of the role of electrochemical processes in thermochemical redox catalysis raises the tantalizing prospect of using spatially resolved electrochemical microscopy techniques to image variations in catalytic activity for a thermochemical transformation.

As current flow can be quantified with excellent sensitivity (noise levels down to tens of femtoamperes), electrochemical microscopy can be used to measure reaction rates across small areas of exposed catalyst with sub-micrometre dimensions. In particular, scanning electrochemical cell microscopy (SECCM) uses an electrolyte-filled pipette to confine the reaction area to the footprint of a contacting droplet bridging the pipette tip and the surface^32–34^; meniscus contact is made to an array of different locations to image electrochemical activity across a catalyst surface. The spatial resolution of the technique is limited primarily by the size of the pipette (typically 30 nm to 2 µm), and imaging from 50 nm^35^ to a few micrometres^36^ resolution has been demonstrated.

SECCM combined with electron backscatter diffraction (EBSD)^33,34,36–38^ is a well-established technique for structure–activity correlations in electrocatalysis. In this study, we extend this powerful approach to the study of thermochemical catalysis. Herein, we develop a workflow and methodology for imaging thermochemical redox catalysis using electrochemical microscopy. The colocated SECCM-EBSD analysis was used to image grain-dependent catalytic activity on polycrystalline Pt for the aerobic oxidation of formic acid (FA). This reaction is known to proceed via the coupling of the electrochemical oxygen reduction reaction (ORR) and FA oxidation reaction (FAOR) half-reactions^25^. As both half-reactions are known to occur with different activity on a wide variety of Pt surface terminations, this reaction provides a stringent test case for the application of this imaging methodology^37,39,40^. We collect electrochemical activity images for each half-reaction in isolation and then corresponding images of catalytic activity when both co-reactants are present. Activity maps of the individual half-reactions in isolation provide initial estimates of the distribution of mixed potentials across the surface during the overall reaction and imply the existence of microscopic lateral current fluxes across the catalyst surface that galvanically couple disparate sites during thermochemical catalysis. Tafel analysis of current–potential profiles in the presence of both reactants provides spatially resolved images of catalytic rate. This analysis also reveals that across short-range length scales, the presence of the co-reactant during thermochemical catalysis serves to alter the current–potential profiles of each constitutive half-reaction relative to those measured in isolation in the absence of the co-reactant. We term this phenomenon chemical cross-talk and find that the presence of O_2_ serves to inhibit or slightly promote the FAOR half-reaction depending on the grain orientation, while the presence of FA serves to substantially attenuate ORR. Both of these chemical cross-talk effects are attributed to the accumulation and removal of poisoning CO species, which are known to be formed during both thermochemical and electrochemical FA oxidation catalysis^41,42^. Overall, this work establishes a methodology for using electrochemical microscopy to image thermochemical catalysis and provides rich insights into how electrochemical half-reactions couple and interact across surface structures to enable thermochemical redox transformations.

## Results

### Grain-dependent FAOR and ORR electrocatalytic activity

**Fig. 1:**
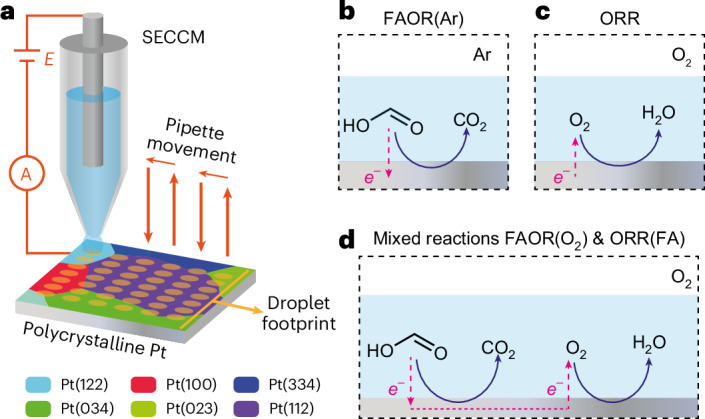
Electrochemical microscopy to image thermochemical catalysis. **a**–**d**, Schematic of the SECCM methodology (**a**) to study each half-reaction in isolation (**b**,**c**) and with both reactions together in the same region of a polycrystalline platinum (Pt) surface (**d**). **b**, Voltammetric measurement of electrochemical FAOR under an argon (Ar) atmosphere in the absence of oxygen (O_2_), FAOR(Ar). **c**, Voltammetric measurement of ORR in the absence of FA. **d**, Voltammetric measurement of FAOR in the presence of O_2_ and ORR in the presence of FA, where FA and O_2_ coexist in the same electrolyte. The areas of electrodes for all three experiments remain the same. Each yellow spot on the Pt surface represents the droplet footprint left by SECCM meniscus contact in each individual measurement. The red arrows indicate the movement of the SECCM pipette from spot to spot across the surface of Pt.

**Fig. 2:**
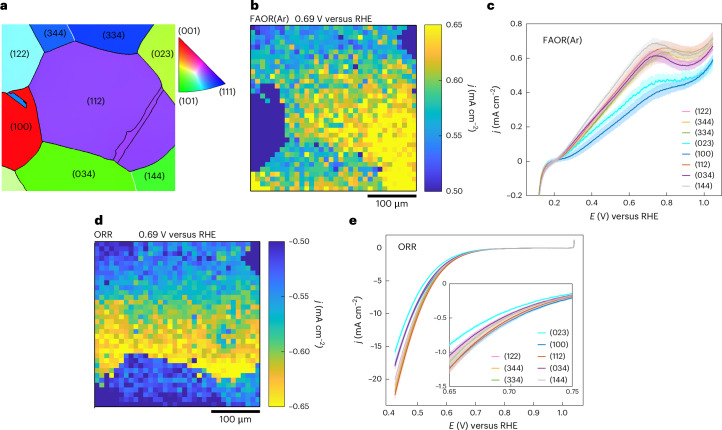
Grain-dependent electrocatalytic activity of isolated half-reactions. **a**, EBSD grain orientation map of the polycrystalline Pt surface probed during the SECCM experiments. **b**, Spatially resolved equipotential snapshot of FAOR(Ar) obtained by SECCM (Supplementary Video 1) at a potential of 0.69 V using 0.5 M FA in 0.1 M HClO_4_ under a continuous Ar flow. **c**, Average voltammograms of eight grains obtained from the FAOR(Ar) measurement. **d**, Spatially resolved equipotential snapshot of ORR obtained by SECCM (Supplementary Video 2) at a potential of 0.69 V using 0.1 M HClO_4_ under a continuous O_2_ flow. **e**, Average voltammograms of eight grains obtained from the ORR measurement. Inset shows the magnified voltammograms in the potential window of 0.65–0.75 V. Scan rates for the LSV curves were 1 V s^−1^. In **c** and **e**, each darker centre line shows the mean current at each potential, and the surrounding lighter band indicates ± standard deviation (s.d.) of the current at each potential. The sample sizes for grains with orientation distributions close to the (122), (344), (334), (023), (100), (112), (034) and (144) planes are *n* = 78, 22, 43, 17, 94, 603, 199 and 44, respectively. The sample size for each orientation corresponds to the cumulative number of SECCM measurements on grains exhibiting that orientation.

As illustrated in Fig. 1, we initially used SECCM to image surface heterogeneity for each half-reaction in isolation: the FAOR under an Ar atmosphere in the absence of O_2_, denoted as FAOR(Ar), and the ORR in the absence of FA. To explain the effect of surface structure, for example, crystallographic orientations, on electrochemical reactions, we used identical-location EBSD. This technique allowed us to characterize the crystallographic structure of the Pt surface, which exhibited eight different grains within the probed region (Fig. 2a) with orientation distributions close to (122), (344), (334), (023), (100), (112), (034) and (144) planes. Notably, the Pt(112) plane consisted of three adjacent grains, all oriented close to this plane as shown in Supplementary Fig. 1. Therefore, we analysed these three grains as a single Pt(112) grain although we acknowledge that there are small differences in surface structure. We subjected the Pt sample to UV and electrochemical cleaning for 300 cycles before each experiment. Supplementary Fig. 2 shows the voltammetric response obtained during the electrochemical cleaning process, with the resulting voltammograms being consistent with a clean polycrystalline Pt surface (hydrogen adsorption and desorption peaks as main indicators)^43^. After rinsing with deionized water and drying with nitrogen gas, we carried out subsequent SECCM measurements on the same 340 × 340 µm^2^ region.

To study the FAOR(Ar), we performed SECCM using a deaerated solution of 0.5 M FA in 0.1 M HClO_4_, with the cell under a continuous Ar gas flow, by recording linear sweep voltammetry (LSV) from 0.10 to 1.05 V versus the reversible hydrogen electrode (RHE) at a scan rate of 1 V s^−1^ at 1,156 positions across the selected area. Leveraging the sensitive current response (at the picoampere level) of SECCM and the conical diffusion regime imposed by the pipette geometry ensures minimal ohmic (*iR*) drop and rapid mass transport^44^. These features permit high voltammetric scan rates, which allows access to a large number of measurements at practical timescales. A Ag/AgCl wire reference electrode was used for SECCM experiments (Methods), and all potentials discussed herein are reported relative to the RHE. The grains of interest spanned sizes from tens to hundreds of micrometres (Fig. 2a). This size range required a 10-µm lateral separation between each SECCM meniscus landing spot to ensure a broad representation of surface grains within a single experiment. We recorded individual LSVs at each position on the surface, which are rendered as equipotential spatially resolved maps (34 × 34 pixels) of current density as a function of potential (Supplementary Video 1). The experiment left footprints derived from the drying of individual SECCM droplet residues as depicted in Supplementary Fig. 3a, with an average diameter of roughly 1.4 µm (Supplementary Fig. 3b). This footprint demarcates the probed area, which was used to calculate the current density. Surface roughness is found to be relatively uniform across all Pt crystal facets and equal to approximately 4 nm (Supplementary Fig. 4). This confirms the equivalency between surface and geometric areas of the SECCM footprint (Supplementary Fig. 5), with a minimal difference of 0.0022–0.0170 µm^2^, leading to a negligible current density increase of 0.14–1.10%.

A qualitative evaluation of the spatially resolved equipotential frame (Fig. 2b, derived from Supplementary Video 1) was captured at 0.69 V. This potential was chosen because it is the average potential at which the currents for FAOR(Ar) and ORR are equal and opposite, providing a predicted estimate of the mixed potential during the overall thermochemical reaction. This analysis reveals grain-dependent FAOR(Ar) activity. This heterogeneity is also visualized from average voltammograms (Fig. 2c) of the eight different grains. To quantitatively assess activity variations, the electrochemical data from each grain are extracted and represented as a distribution of current densities at 0.69 V (Supplementary Fig. 3c), which also reflects the surface heterogeneities within individual grains. We observed average current densities as high as 0.66 ± 0.02 mA cm^−2^ in the Pt(144) grain (yellow in Fig. 2b) and in some regions of Pt(112), which exhibited 0.61 ± 0.04 mA cm^−2^. In contrast, current densities as low as 0.36 ± 0.03 mA cm^−2^ were observed for Pt(100). Within each grain, current density distributions showed some heterogeneity (standard deviations of 0.02–0.04 mA cm^−2^) that can be attributed to the subtle surface structural variations within grains rather than potential artefacts from directional scanning of the SECCM pipette (Supplementary Fig. 6). Through an inverse pole figure representation (Supplementary Fig. 3d), we noted a general trend where grains with orientations closer to (100) were less active for FAOR(Ar). This observation aligns with previous single crystal measurements where Pt(100) was less reactive unless pretreated with oxygen, whereas both Pt(111) and Pt(110) readily catalyse FA oxidation^45^.

To study the ORR half-reaction, we performed a SECCM experiment using a 0.1 M HClO_4_ solution under a continuous O_2_ gas flow, and recorded LSVs from 1.04 to 0.42 V versus RHE at a scan rate of 1 V s^−1^ over the same selected area (Fig. 2a and Supplementary Fig. 7a), applying the same cleaning procedure. The dynamic spatially resolved response is represented as a sequence of current density maps as a function of potential (Supplementary Video 2). The average diameter of the footprints was roughly 1.3 µm in this case (Supplementary Fig. 7b). The equipotential map captured at 0.69 V (Fig. 2d) along with the average voltammograms (Fig. 2e) display the heterogeneous grain-dependent ORR activities. The distribution of current densities at 0.69 V (Supplementary Fig. 7c) reveals that grains with orientations close to (100) and (112) exhibit superior ORR activity (0.62 ± 0.03 mA cm^−2^ and 0.59 ± 0.04 mA cm^−2^, respectively), while Pt(023) is the less active (0.44 ± 0.02 mA cm^−2^) among the 8 grain orientations. The inverse pole figure (Supplementary Fig. 7d) shows that grain orientations with contributions from (111) and (100) exhibited higher activity compared to those with contributions from (110) and (100), a finding that aligns with previous SECCM measurements^37^.

### Inter-grain cooperativity during thermochemical catalysis

Mixed potential theory has been demonstrated to accurately describe the correlation between an overall thermocatalytic aerobic oxidation and its constituent electrochemical half-reactions for carbon-supported metal nanoparticle catalysts^25^. Within this framework, coupled catalytic active sites operate as a short-circuited electrochemical cell, where both half-reactions occur, generating an equal yet opposite current flux that is maintained at a steady-state mixed potential^25,26^. Our previous research confirmed the validity of this short-circuit model and specifically showed that for carbon-supported Pt nanoparticles^25^, the mixed potential and reaction rate are well predicted by simply overlaying the current–potential curves for each individual half-reaction. In these previous studies, the conductive carbon support affords facile electrochemical coupling of half-reactions occurring on disparate particles and the Pt nanoparticles themselves feature a diversity of facets and surface defects capable of electrochemical coupling with each other. As a consequence, these previous studies obscure the nuances of electrochemical coupling on short length scales localized to a single surface termination. To unpack this complexity, we first apply mixed potential theory to the electrochemical measurements of each half-reaction in isolation to arrive at a prediction of the rate and mixed potential during thermochemical catalysis and then compare these predictions to the measured values of mixed potential and rate in the presence of both reactants in the following sections.

**Fig. 3:**
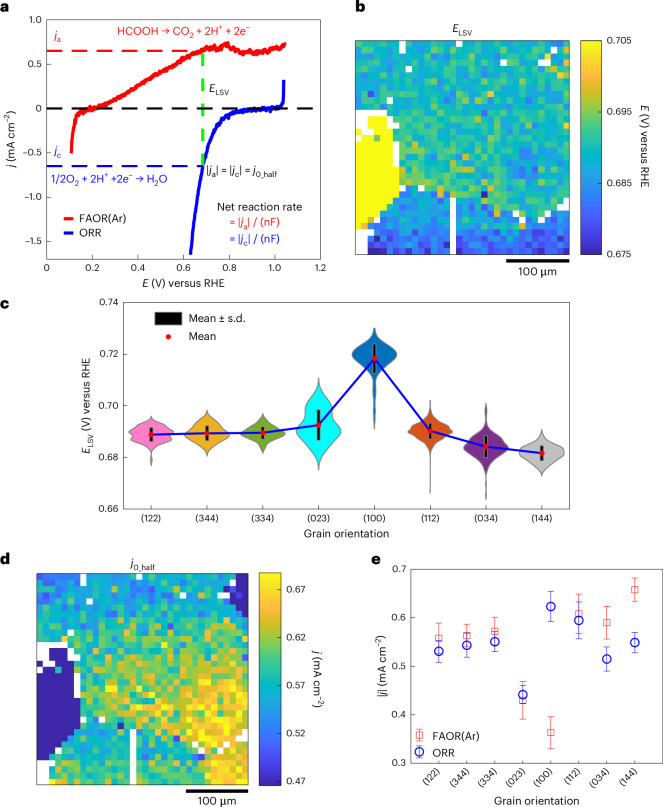
Predicted mixed potential and inter-grain cooperativity. **a**, Representative LSV traces of isolated FAOR(Ar) and ORR illustrating the calculation of the current equivalency potential (*E*_LSV_) and the mixed current density (*j*_0_half_), where the absolute current densities of the cathodic (*j*_c_) and anodic (*j*_a_) reactions are equal. **b**, *E*_LSV_ map produced from all SECCM spots, based on the mentioned calculation. **c**, The violin plot of *E*_LSV_ on the eight different Pt grains probed during the SECCM experiments. **d**, *j*_0_half_ map at *E*_LSV_ produced from all SECCM spots. Note that some pixels in these maps were left blank (white) to account for a small lateral shift between corresponding pixels for the two independent SECCM experiments, particularly to avoid mismatch in pixels close to grain boundaries. **e**, Grain-average surface current density (absolute value) at 0.69 V for FAOR(Ar) and ORR. The error bars in **c** and **e** represent ± s.d. from the mean. The sample sizes for grains with orientation distributions close to the (122), (344), (334), (023), (100), (112), (034) and (144) planes are *n* = 78, 22, 43, 17, 94, 603, 199 and 44, respectively. The sample size for each orientation corresponds to the cumulative number of SECCM measurements on grains exhibiting that orientation.

Figure 3a illustrates that by overlaying the LSV transients from representative SECCM pixels of FAOR(Ar) and ORR, after the capacitive current correction (with a negligible value of 0.02 ± 0.06 mA cm^−2^ for FAOR(Ar) and 0.06 ± 0.004 mA cm^−2^ for ORR, respectively), a predicted current equivalency point (*E*_LSV_) can be obtained, where the current densities of the independent half-reactions are equal in magnitude, but opposite in sign. Applying this analysis to all the corresponding SECCM pixels of FAOR(Ar) and ORR reveals grain-dependent behaviour, as shown in Fig. 3b,c. From the grain-average values (blue line, Supplementary Fig. 8a), an *E*_LSV_ as high as 718 ± 6 mV is observed for grain (100), while the lowest value (682 ± 3 mV) is observed for grain (144). Through the mixed current from the individual half-reactions (*j*_0_half_) map (Fig. 3d) and the grain-average *j*_0_half_ (red line, Supplementary Fig. 8a), we can derive a prediction of the grain-dependent reaction rate for the FA thermochemical catalytic oxidation in the limit that the half-reactions operate independently. The results show that Pt(100) provides the lowest *j*_0_half_ (0.38 ± 0.03 mA cm^−2^), with Pt(023) being the second lowest (0.43 ± 0.04 mA cm^−2^), suggesting that these orientations are less active for the thermochemical oxidation. Meanwhile, Pt(112) and Pt(144) are the most active grain orientations (0.61 ± 0.04 and 0.65 ± 0.02 mA cm^−2^, respectively). Notably, the grain-dependent behaviour of *j*_0_half_ (Fig. 3d) is diminished relative to the grain-dependence for each individual half-reaction (Fig. 3e). This is because *j*_0_half_ samples the activity for both FAOR(Ar) and ORR. Thus, a grain orientation that displays high activity for one half-reaction may display counteractingly lower activity for the other half-reaction, leading to similar activity for the overall reaction, *j*_0_half_. The inverse pole figures (Supplementary Fig. 8b,c) show that grains with greater contribution from Pt(100) generally exhibit a more positive *E*_LSV_ and lower *j*_0_half_. The average value (691 ± 10 mV) of *E*_LSV_ from all the SECCM measurements (Fig. 3b) is close to the corresponding *E*_LSV_ value of 0.75 V measured for ensemble-averaged carbon-supported Pt nanoparticles^25^.

The differences in *E*_LSV_ for the different regions of the catalyst are reflective of the differences in relative activity towards the two half-reactions. Specifically, grain orientations with a more positive *E*_LSV_ reflect a higher activity for ORR relative to FAOR while a more negative *E*_LSV_ reflects a higher activity for FAOR relative to ORR. These differences in *E*_LSV_ highlight a key principle describing the action of the catalyst ensemble. In particular, when an ensemble of surface terminations is exposed to a common electrolyte media in bulk thermochemical catalysis, the active sites at each surface termination are electronically and ionically connected to each other; thus, disparate grains will galvanically couple to each other driven by differences in their individual *E*_LSV_ values, as all electronically conductive sites that share the same electrolyte medium must adopt the same potential. For the grains to adopt the same potential, there must be lateral current flow between sites of differing activity. A possible experiment in which such galvanic coupling could be directly observed would entail connecting two separated Pt grains via an external wire and measuring the current flow between the grains. Comparing the spatially resolved equipotential frames at 0.69 V for both FAOR(Ar) (Fig. 2b) and ORR (Fig. 2d) reveals substantial variations (Fig. 3e) in relative activity between the two half-reactions from grain to grain. The potential of 0.69 V is chosen because it is the average predicted mixed potential (Fig. 3b,c). For many of the grains with orientations close to (122), (344), (334), (023) and (112), we observe similar average current densities at 0.69 V towards FAOR(Ar) and ORR, with a maximum difference of 5% (Supplementary Table 1). However, FAOR(Ar) and ORR activities diverge for other grains. For example, grains close to (034) and (144) show about 14.5% and 19.9% higher current density towards FAOR(Ar) than ORR, respectively. The most substantial reactivity difference is observed on Pt(100), which is more active for ORR than FAOR(Ar), displaying roughly 42% higher average current density towards ORR than FAOR(Ar).

Considering these findings from a thermochemical catalysis perspective, wherein the catalyst surface acts as a unified assembly of distinct grains, our results indicate an interplay between different microscale active sites for the FAOR(Ar) and the coupled ORR across the full ensemble of surface terminations. This interplay indicates the existence of lateral charge flow across the most active sites, creating a galvanic coupling between different regions. Regions of different orientations will form short-circuited electrochemical cells with one another. To achieve the uniform potential required by the electrical and ionic conductivity of the system, regions with relatively positive isolated mixed potentials will be driven to become cathodes and regions with relatively negative isolated mixed potentials will be driven to become anodes. Because each region is thus driven to carry out in excess the reaction for which it is relatively more active, this phenomenon is cooperative in nature and may lead to greater overall thermochemical activity for the ensemble than for each isolated grain. The magnitude of this rate enhancement depends on the magnitude of the disparity between the relative rates of anodic and cathodic half-reactions at the different grains. This analysis also challenges the commonplace notion in thermochemical heterogeneous catalysis that a singular facet or site is the most active for a given reaction and suggests instead that the coupling of disparate sites and facets may furnish the highest reaction rates.

### Local mixed potentials and catalytic rates

**Fig. 4:**
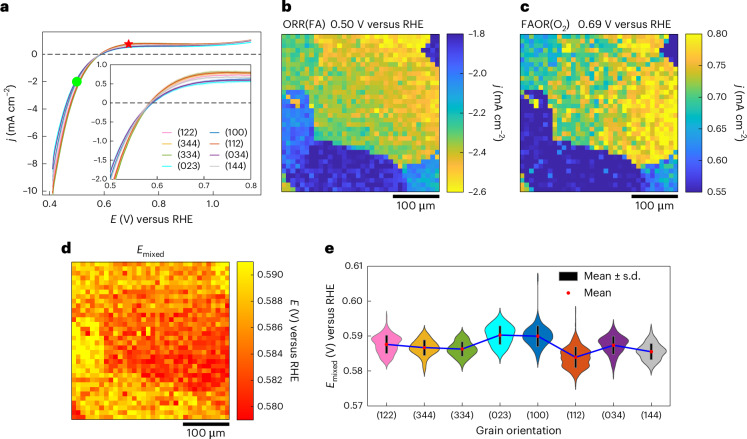
Grain-dependent measured mixed potentials. **a**, Average voltammograms of the eight different Pt grains obtained from the SECCM measurement using 0.5 M FA in 0.1 M HClO_4_ under a continuous O_2_ flow, at a scan rate of 1 V s^−^^1^. **b**,**c**, Spatially resolved equipotential snapshots obtained by SECCM (Supplementary Video 3) at potentials of 0.50 (**b**) and 0.69 V (**c**), corresponding to the locations marked by green circle and red star in **a**, respectively. **d**, Spatially resolved mixed potential (*E*_mixed_) map, built using the potential value where the net current equals zero. **e**, Violin plot of *E*_mixed_ for the eight different Pt grains probed by SECCM. In **a**, each darker centre line shows the mean current at each potential, and the surrounding lighter band indicates ±s.d. of the current at each potential. The error bars in **e** represent ±s.d. (black bar) from the mean (red dot). The sample sizes for grains with orientation distributions close to the (122), (344), (334), (023), (100), (112), (034) and (144) planes are *n* = 86, 24, 42, 17, 95, 650, 212 and 47, respectively. The sample size for each orientation corresponds to the cumulative number of SECCM measurements on grains exhibiting that orientation.

To study how the surface structure affects the measured mixed potential and catalytic rate, we carried out an SECCM experiment using 0.5 M FA in 0.1 M HClO_4_ under a continuous O_2_ gas flow over the same area as previous experiments. In this case, the voltammetric sweep was from 0.40 V to 1.14 V at a scan rate of 1 V s^−1^. This large potential range captures regions at low potentials where the observed current is dominated by ORR even while in the presence of FA (ORR(FA)) and regions at positive potential where the observed current is dominated by FAOR even while in the presence of O_2_ (FAOR(O_2_)). At intermediate potentials, the observed current is the sum of countervailing contributions from ORR(FA) and FAOR(O_2_). These different regions can be observed from the average voltammograms (Fig. 4a) of 8 different grains, and the spatially resolved maps for ORR(FA) taken at 0.50 V (Fig. 4a, green circle) and FAOR(O_2_) taken at 0.69 V (Fig. 4a, red star) are displayed in Fig. 4b,c, respectively. The spatially resolved electrochemical video is represented in Supplementary Video 3, and examples of the footprints left by the SECCM experiment (roughly 1.4 µm) are shown in Supplementary Fig. 9a,b. These activity maps reveal that strong grain-dependent activity variations persist even when both reactants are present in the media. As observed above for FAOR(Ar) and ORR recorded independently, many grains display comparable activity for ORR(FA) as well as FAOR(O_2_) leading to the similarity in the overall map in Fig. 4b,c.

The zero crossing of the voltammetric traces provides a direct measurement of the mixed potential, *E*_mixed_, at which FAOR(O_2_) and ORR(FA) display equal and opposite current densities, resulting in a net current of zero. To analyse the grain-dependence of *E*_mixed_ across the Pt surface, we extracted the *E*_mixed_ in each SECCM pixel to create a spatially resolved map (Fig. 4d). The distribution of (Fig. 4e) and grain-averaged (blue line in Supplementary Fig. 9c,d) *E*_mixed_ values show more positive values on Pt(023) and Pt(100), 590 ± 3 mV, and a lower *E*_mixed_ (584 ± 3 mV) was observed on Pt(112). Further SECCM experiments (Supplementary Note 1) conducted on another Pt surface (Supplementary Fig. 10) at a slower scan rate (200 mV s^−1^), also reveal similar grain-dependence for FAOR(Ar), ORR and FAOR(O_2_) activity (Supplementary Figs. 11 and 12). We note that the mean *E*_mixed_ values fall in the range of 584 mV to 590 mV, which is approximately ~0.1 V lower than the range of *E*_LSV_ values predicted from electrochemical data of each half-reaction in isolation: this topic is discussed in detail below. Notwithstanding, the differences in *E*_mixed_ across the probed surface are relatively small, with a maximum average difference of 6 mV. This is a direct reflection of the fact that for most grains, high FAOR(O_2_) activity also coincides to high ORR(FA) activity, as seen in Fig. 4b,c. Thus, while an increased rate of FAOR(O_2_) for a given grain will push *E*_mixed_ to lower values, this is compensated for on most grains by a coincident acceleration in the rate of ORR(FA) that pushes *E*_mixed_ to higher values. The compensation between the two effects lead to *E*_mixed_ values that are relatively similar across grains. Nonetheless, we stress that given the metallic conductivity of the support and the short length scales separating grains, these small variations in *E*_mixed_ are more than ample to drive lateral current flows between grains, or between hot spots on any given grain. In addition, the ability to quantify these small variations in *E*_mixed_ highlights the sensitivity of this technique for catalytic imaging.

**Fig. 5:**
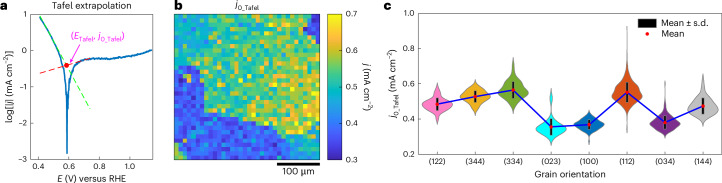
Grain-dependent catalytic rates. **a**, Representative voltammogram in Tafel representation (log|*j*|−*E* plot) used for calculating the mixed current density (*j*_0_Tafel_) for the ORR (with presence of FA) and the FAOR (with presence of O_2_). This representative plot displays *E*_mixed_ (0.589 V), *E*_Tafel_ (0.585 V), FAOR Tafel slope (735 mV dec^−1^), ORR Tafel slope (128 mV dec^−1^) and *j*_0_Tafel_ (0.393 mA cm^−2^). **b**,**c**, SECCM spatially resolved map (**b**) and grain-dependent violin plot (**c**) of *j*_0_Tafel_ calculated from the Tafel analysis. The error bars in **c** represent ±s.d. (black bar) from the mean (red dot). The sample sizes for grains with orientation distributions close to the (122), (344), (334), (023), (100), (112), (034) and (144) planes are *n* = 86, 24, 42, 17, 95, 650, 212 and 47, respectively. The sample size for each orientation corresponds to the cumulative number of SECCM measurements on grains exhibiting that orientation.

At the mixed potential, *E*_mixed_, the net current is zero, but the thermochemical catalytic rate can, nonetheless, be estimated by fitting the voltammetric data positive and negative of *E*_mixed_. This same method of Tafel analysis was recently applied to analyse aggregate catalytic activity of Pd/C and Au/C catalysts^46^. This Tafel analysis is graphically illustrated in Fig. 5a. The fit of the ORR(FA)-dominated curve negative of mixed potential is shown in Fig. 5a (green dashed line) and the fit of the FAOR(O_2_)-dominated curve positive of the mixed potential is shown in Fig. 5a (red dashed line). These two fits intersect with each other at *E*_Tafel_, to furnish, *j*_0_Tafel_, a direct estimate of the cross-current flowing between the two half-reactions during overall thermochemical catalysis. While the potential independence of the FAOR data positive of *E*_mixed_ might seem to imply transport limitations, calculations of transport-limited FAOR current in the SECCM droplet (Supplementary Fig. 13) return values 2–3 orders of magnitude higher than the observed currents (see Supplementary Section 2 for calculation details). Thus, the observed current plateau is not due to transport artefacts, but is instead attributed to CO poisoning, a well-documented phenomenon for FAOR on Pt^42^. In the limit that the Tafel regions of the fit are reflective of the kinetics of the half-reactions at the mixed potential, the intersection point *E*_Tafel_ should match *E*_mixed_ and indeed, we observe that these two values match to within 1–4 mV across all the spots investigated (Supplementary Fig. 14). This correspondence further supports the validity of this analysis. Accounting for the electron stoichiometry and Faraday’s constant, these cross currents directly correspond to the local catalytic rate of thermochemical FA oxidation catalysis. The spatially resolved map (Fig. 5b) and distributions (Fig. 5c) of *j*_0_Tafel_ clearly reveal the grain-dependent variation in thermochemical reaction rates. The ORR(FA) Tafel slopes across all spots in the 0.44–0.54 V potential range are shown in Supplementary Fig. 15a, and the FAOR(O_2_) Tafel slope in the 0.65–0.75 V potential range, which is free from mass-transport limitation, is shown in Supplementary Fig. 15b. By calculating the average *j*_0_Tafel_ (red line in Supplementary Fig. 9c and Supplementary Fig. 9e) as a function of grain orientations, we observe that Pt(023), Pt(100) and Pt(034) show relatively low *j*_0_Tafel_ (0.36 ± 0.05 mA cm^−2^, 0.37 ± 0.03 mA cm^−2^ and 0.38 ± 0.04 mA cm^−2^, respectively). In contrast, Pt(334) displays a relatively high activity (*j*_0_Tafel_ of 0.56 ± 0.05 mA cm^−2^). The variability in activity observed among different grains during simultaneously occurring half-reactions demonstrates that even if the current flow required to galvanically couple both electrochemical half-reactions is confined to short distances within individual grains, there will nonetheless be spatial heterogeneity in local reaction rates for the thermochemical FA oxidation across the catalyst surface. These measurements of local catalytic activity are inherently indirect because it is difficult, if not impossible, to directly quantify product formation by conventional methods (for example, gas chromatography) on this length scale, given the small amount of product generated within each spot. However, at the slower scan rate of 200 mV s^−1^, the average SECCM-inferred catalytic rate across the four distinct grains (Supplementary Fig. 10), 0.04 ± 0.01 mA cm^−2^, is roughly in line with the specific rate of thermochemical FA oxidation (Supplementary Fig. 16), 0.012 ± 0.001 mA cm^−2^, measured independently by gas chromatography quantification of the produced CO_2_ from a polycrystalline Pt foil under the same reaction conditions (Supplementary Note 3). Thus, albeit indirect, this analysis provides among the highest resolution maps of local activity for a thermochemical catalytic reaction, highlighting the power of this electrochemical imaging methodology.

### Origin of chemical cross-talk between co-reactants

**Fig. 6:**
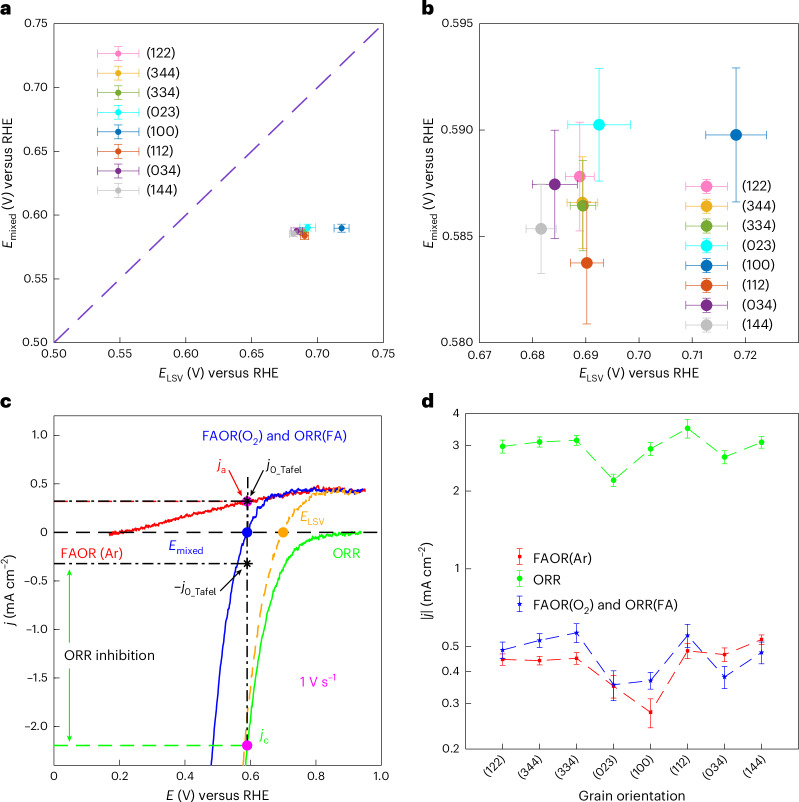
Origin of chemical cross-talk between co-reactants. **a**, Relationship between the average equivalent current potential (*E*_LSV_) and the average mixed potential (*E*_mixed_) on the eight different Pt grains probed with SECCM. Dashed line indicates perfect correspondence. **b**, Magnified version of the plot in **a**. **c**, Schematic illustrating the calculation of the current density for FAOR(Ar) and ORR at measured *E*_mixed_ at 1 V s^−1^. **d**, Average mixed current densities for FAOR(O_2_) and ORR(FA) based on Tafel analysis, and current densities extracted at *E*_mixed_ for FAOR(Ar) and ORR as a function of Pt grains. The error bars represent the standard deviation from the mean, based on measurements from each different grain. The error bars in **a** and **b** represent ±s.d. from the mean of both *E*_mixed_ and *E*_LSV_. The error bars in **d** represent ±s.d. from the mean. The sample sizes of *E*_mixed_ (**a**,**b**) and average mixed current densities (**d**, blue data) for grains with orientation distributions close to the (122), (344), (334), (023), (100), (112), (034) and (144) planes are *n* = 86, 24, 42, 17, 95, 650, 212 and 47, respectively. The sample sizes of average mixed current densities (**d**, red and green data) for grains with orientation distributions close to the (122), (344), (334), (023), (100), (112), (034) and (144) planes are *n* = 78, 22, 43, 17, 94, 603, 199 and 44, respectively. The sample size for each orientation corresponds to the cumulative number of SECCM measurements on grains exhibiting that orientation.

The above studies allowed us to determine the current equivalency potential (*E*_LSV_) derived from analysis of each independent half-reaction in isolation, and the measured mixed potential (*E*_mixed_) derived from the zero current potential when both reactions take place simultaneously. In the limit that the two half-reactions are entirely independent of each other, the *E*_LSV_ should match the *E*_mixed_ during the net thermochemical FAOR (purple dashed parity line, Fig. 6a), and the associated *j*_0_half_ should match *j*_0_Tafel_ (purple dashed parity line, Supplementary Fig. 17a). Notably, in this ideal case, *j*_0_ also corresponds to the overall thermochemical reaction rate measured independently^25,26,28^. However, the data presented in Fig. 6a,b indicate a deviation from the predicted values, with the measured *E*_mixed_ (~0.6 V) systematically lower than the predicted *E*_LSV_ (~0.7 V) values by ~0.1 V. This perturbation is also reflected in deviations, albeit to a smaller extent, between the mixed current density *j*_0_half_ and *j*_0_Tafel_ (Supplementary Fig. 17) for many of the grains. This observation implies that when the reaction is confined to the small length scales and singular metal facets probed here, the presence of O_2_ perturbs the electrokinetic profile of electrochemical FA oxidation and/or vice versa. We define this effect as chemical cross-talk between the co-reactants and the independent half-reactions, that is, O_2_ influences the FAOR and FA affects the ORR.

To further probe the bases for the above discrepancy, we compared the aggregate voltammetric profile when both reactants are present (Fig. 6c, blue) in the same electrochemical cell with the voltammetric profile of FAOR(Ar) (Fig. 6c, red) and ORR (Fig. 6c, green) in isolation. In addition, we mathematically summed the isolated half-reaction data to produce a simulated voltammetric profile (Fig. 6c, orange dotted) in the limit that the two half-reactions are unperturbed by the presence of the co-reactant. For this representative overlay, we observed a ~0.1 V negative shift of the observed *E*_mixed_ (Fig. 6c, blue dot) and the predicted *E*_LSV_ (Fig. 6c, orange dot). While the combined LSV with both reactants (Fig. 6c, blue) begins to overlay with the FAOR(Ar) (Fig. 6c, red) data at potentials slightly positive of *E*_mixed_, the combined LSV is always negative of the ORR data in isolation (Fig. 6c, green) even at potentials well negative of *E*_mixed_. This analysis highlights that chemical cross-talk substantially attenuates ORR but has a far more modest effect on FAOR.

The above qualitative observation can be further quantified by comparing the expected current densities for FAOR(Ar) and ORR in the hypothetical absence of chemical cross-talk (Fig. 6c, pink dots) at the observed *E*_mixed_ with the inferred rate (Fig. 6c, black star points) of catalysis, *j*_0_Tafel_, from the above Tafel analysis. This analysis reveals that the average *j*_0_Tafel_ for the combined reaction, FAOR(O_2_), is slightly higher than the current density at *E*_mixed_ for the FAOR(Ar) in most grains (blue versus red data, Fig. 6d), although the absolute differences are small, ranging from 0.01 mA cm^−2^ to 0.11 mA cm^−2^ (Supplementary Table 2). This indicates that the presence of O_2_ slightly promotes the FAOR half-reaction on most grains^47^, with the largest effect on Pt(100) where the FAOR rate enhanced by a factor of 1.32 (Supplementary Fig. 18 and Supplementary Table 2), probably due to the facilitated removal of adsorbed CO—a known poison for FAOR^41,48^—formed under the 1 V s^−1^ scan rate condition. As noted qualitatively above, this analysis quantifies the substantial attenuation of ORR in the presence of FA. The average *j*_0_Tafel_ for ORR(FA) is 1.84 mA cm^−2^ to 2.95 mA cm^−2^ (Supplementary Table 2) lower than the corresponding values for ORR in isolation and this inhibition is observed across all grains (blue versus green data in Fig. 6d, and Supplementary Fig. 18) with ORR inhibited to the largest extent on Pt(100) by a factor of 8. This strong attenuation of ORR is also attributed to the generation of CO poisons that form during concurrent FAOR^49,50^. The poisoning effect of CO serves to increase the required overpotential for ORR to achieve a given rate leading to the observed negative shift of *E*_mixed_ relative to *E*_LSV_.

Comparison of the results obtained at scan rates of 1 V s^−1^ and 200 mV s^−1^ reveals that the qualitative variations across grains are largely preserved across different scan rates (Supplementary Figs. 11 and 12). However, at lower scan rates, the reaction is more susceptible to poisoning on average, as evidenced by an attenuation in *j*_0_Tafel_ across all grains (Supplementary Fig. 12e). This suggests that under severe CO poisoning at lower scan rates, oxygen is insufficient to remove the adsorbed CO, as excessive CO coverage can hinder the formation of oxygen-containing species necessary for its oxidation^51^.

Notably, the negative shift of *E*_mixed_ relative to *E*_LSV_ is not observed for Pt nanoparticles supported on carbon^25^, a phenomenon that may arise from many factors including metal support interactions, distinct facets or defects present on nanoparticles, and/or gradients in O_2_ and/or formate within the Pt/C catalyst film. In addition, due to the inter-grain coupling, the observed activity on a bulk catalyst surface may be less susceptible to poisoning from chemical cross-talk than the individually measured SECCM spots, as sites that are poisoned for a given half-reaction can couple to sites that are unpoisoned for the same half-reaction such that the bulk activity is higher than what would be expected by measuring individual grains in isolation. All these effects may serve to enhance the oxidative removal of CO poisons^47,50,52^ and/or to inhibit the degree of chemical cross-talk in the first place. Notwithstanding, these findings suggest a potential benefit to spatially separating ORR and FAOR catalysis, perhaps via a mixed ion–electron conducting membrane, so as to minimize chemical cross-talk and maximize catalytic rates.

## Conclusions

In this study, we demonstrate that SECCM-EBSD can be used to generate spatially resolved images of local catalytic rates for thermochemical redox transformations that proceed via the coupling of electrochemical half-reactions. Using FA oxidation as a test case, we analyse the current–potential profile of oxygen reduction and FA oxidation in isolation and the aggregate profile in the presence of both co-reactants. Analysis of each half-reaction in isolation reveals grain-dependent variations in catalytic activity. While many grains display similarly high (or low) activity for both half-reactions, some grains, such as Pt(100), display divergent activity for each half-reaction with high activity for ORR and low activity for FAOR(Ar). These findings challenge the notion of a single most active surface site and instead imply inter-grain cooperativity during ensemble thermochemical catalysis via lateral current flows that galvanically couple grains with preferential competency for each half-reaction. Analysis of the aggregate current–potential profile in the presence of O_2_ and FA reveals that strong grain-dependent activity variations persist even when the half-reactions take place concurrently and Tafel analysis provides direct images of local variations in catalytic rate with high spatial resolution. Finally, comparison of the isolated and aggregate current–potential profile reveals the nature of chemical cross-talk between half-reactions. The presence of FAOR serves to substantially attenuate ORR activity, an effect that is ascribed to the presence of CO poisons on the catalytic surface. Overall, this study builds a bridge between electrochemical microscopy and thermochemical catalysis and showcases how electrochemical half-reactions couple and interact across surface structures to enable redox transformations.

## Methods

### Sample preparation

A platinum foil (99.95+%, Goodfellow) with a thickness of 0.25 mm was manually polished using a polishing cloth (MicroCloth, Buehler) and monocrystalline diamond water-soluble sprays (Kemet) of various particle sizes: 3 µm, 1 µm, 0.5 µm and 0.1 µm, respectively. After each polishing step, the Pt foil was sonicated in deionized water (18.2 MΩ cm^−1^ at 25 °C, ELGA Labwater) for 30 min to remove any remaining diamond powder. Before being used as a working electrode, the Pt foil was annealed under a butane flame and then quickly quenched in deionized water, a process that was repeated 3–5 times. This step was important for cleaning surface contaminants, alleviating surface pressure, and allowing the surface to recrystallize to show clear grains and grain boundaries. Before each SECCM measurement, the Pt foil was first cleaned using a UV cleaner (Zone II for scanning electron microscopy, Hitachi) to eliminate hydrocarbon contamination from electron microscopy imaging. The Pt foil was then electrochemically cleaned in a standard three-electrode system, using a Pt coil as the counter electrode and a commercial leakless Ag/AgCl electrode (3.4 M KCl, LF-2-100, Innovative Instruments, Inc.) as the reference electrode. Cyclic voltammetric measurements were performed on the Pt foil in deaerated 0.1 M HClO_4_. The scan window was −0.2 V to 1.0 V, and each CV cycle started and stopped at 0.15 V, versus Ag/AgCl, beginning each cycle with scanning to positive potentials. This process was repeated for 300 cycles at a scan rate of 50 mV s^−1^. The Pt foil was then rinsed with deionized water and dried under N_2_ gas flow in preparation for the local SECCM experiments. These electrochemical and UV cleaning processes did not substantially change the Pt surface structure as analysed by EBSD (Supplementary Fig. 19).

### Pipette with electrolyte and quasi-reference electrode

Single-barrel pipettes with a diameter of 1 µm were prepared using a P2000 laser-based micropipette puller (Sutter Instruments) from borosilicate capillaries (1.2-mm outer diameter, 0.69-mm inner diameter, 100-mm length, BF120-69-10, Harvard Apparatus). An Ag wire (Goodfellow,) with a diameter of 0.125 mm was electrochemically oxidized within a potential window between +5 V and +7 V versus a Pt counter electrode in a saturated KCl solution for 10–15 min to prepare an AgCl-coated Ag quasi-reference counter electrode (QRCE). The Ag/AgCl QRCE, demonstrating high stability^53^, was routinely calibrated by measuring the open circuit potential with respect to a commercial leakless Ag/AgCl electrode both before and immediately after each SECCM experiment. This potential was then referenced to the RHE. The SECCM pipettes were filled with different solutions depending on the process being studied: deaerated 0.5 M FA in 0.1 M HClO_4_ for the FAOR(Ar), 0.1 M HClO_4_ for the ORR and 0.5 M FA in 0.1 M HClO_4_ for the FAOR–ORR mixed processes.

### SECCM experiments

SECCM experiments were conducted using a custom-built multifunctional instrument, as described previously^33,36^. The SECCM setup was placed inside a Faraday cage with heat sinks and acoustic foam to minimize electrical noise, thermal drift and acoustic noise. The Faraday cage was placed on an optical table (RS 2000, Newport) to minimize mechanical vibrations. The system was controlled using LabVIEW 2019 software, running the Warwick Electrochemical Scanning Probe Microscopy platform (http://www.warwick.ac.uk/electrochemistry/wec-spm), in communication with a field-programmable gate array card (PCIe-7852R, National Instruments) for data acquisition and instrument control. The current was measured every 4 µs and averaged 257 times (1 point for data transfer), yielding a data acquisition rate of 1,028 µs per data point.

The Pt foil, which served as the working electrode, was placed inside an environmental cell^54^ under a continuous flow of gas (either Ar or O_2_). This cell was on top of an *xy* piezoelectric positioner (P-733.2, Physik Instrumente) for lateral movement. The pipette was filled with the required electrolyte, equipped with an Ag/AgCl QRCE, and then mounted on a *z*-piezoelectric positioner (P-753.2, Physik Instrumente). Micropositioners (M-461-XYZ-M, Newport) provided coarse movement in *x*, *y* and *z* axes to bring the pipette close to the Pt surface. Hopping-mode SECCM was used to perform experiments on the Pt surface. The pipette initially approached the surface under bias potentials of −0.45 V, 0.50 V and −0.13 V versus the Ag/AgCl QRCE for FAOR(Ar), ORR and mixed reactions, respectively, until the liquid meniscus at the tip of the pipette contacted the Pt surface. The surface current was monitored and used as a feedback signal to stop the vertical movement of pipette when contact was detected. LSV measurements were then carried out using different potential windows and sweep directions depending on the reaction studied. A scan rate of 1 V s^−1^ was used to access thousands of measurements across the surface at a practical timescale. After measurements at one location were finished, the pipette was retracted and moved to the next location across the Pt surface with a lateral distance of 10 μm to repeat the same measurement protocol. The pipette movement follows a single, consistent direction, as illustrated in Supplementary Fig. 6a, to eliminate any potential activity differences arising from scan direction, as demonstrated in Supplementary Fig. 6b.

### Physical characterization

The footprints left by SECCM were imaged by field emission scanning electron microscopy (ZEISS Gemini) to determine the geometric surface area and calculate current densities. A JEOL 7800F with a symmetry EBSD (Oxford Instruments) detector was used to identify the grain orientations of the identical SECCM locations. EBSD data were analysed using AztecCrystal software. The Pt topography was characterized by atomic force microscopy (Park NX10) to evaluate the surface roughness (root mean-square roughness, *R*_q_).

### Data analysis

All the raw data were processed with MATLAB. For FAOR(Ar), a small plateau current appears in the double-layer capacitance region around 0.2 V, and the capacitance was corrected by extracting the current from this plateau and shifting the voltammograms downward by the corresponding value. For ORR, a plateau current is observed at high potentials, and the capacitance was corrected by directly moving this plateau to zero. For FAOR(O_2_), only the stray capacitance was corrected, as no plateau was available to determine a specific value for capacitance adjustment.

In our analysis to predict the current equivalency point (*E*_LSV_), individual SECCM pixels from different experiments were matched to correspond to near identical locations. However, achieving exact pixel-by-pixel alignment in repeated SECCM experiments is challenging, potentially resulting in minor mismatches between pixels in these studies (albeit less than 10 µm). To mitigate the effects of these discrepancies, particularly around grain boundaries, we have excluded certain pixels from the maps (Fig. 3b,d) and corresponding analyses. This approach ensures that all corresponding data points align with the same grain and their proximate locations for all the independent experiments. Inverse pole figures were made following a previously reported methodology^38^. Tafel analysis was performed using a Python code specifically tailored for the data in this study^55^.

## Supplementary information

Supplementary Information Supplementary Figs. 1–19, Notes 1–3, Tables 1 and 2 and References.

Supplementary Video 1 SECCM electrochemical video for FAOR(Ar) under a continuous Ar gas flow at a scan rate of 1 V s^−1^.

Supplementary Video 2 SECCM electrochemical video for ORR under a continuous O_2_ gas flow at a scan rate of 1 V s^−1^.

Supplementary Video 3 SECCM electrochemical video for FAOR under a continuous O_2_ gas flow at a scan rate of 1 V s^−1^.

Supplementary Video 4 SECCM electrochemical video for FAOR(Ar) under a continuous Ar gas flow at a scan rate of 200 mV s^−1^.

Supplementary Video 5 SECCM electrochemical video for ORR under a continuous O_2_ gas flow at a scan rate of 200 mV s^−1^.

Supplementary Video 6 SECCM electrochemical video for FAOR under a continuous O_2_ gas flow at a scan rate of 200 mV s^−1^.

## Data Availability

The main data supporting the findings of this study are available within the paper and related Supplementary Information. The source data for the figures is available via Zenodo at 10.5281/zenodo.16749175 (ref. ^56^). All other data are available from the authors upon reasonable request.
