# Supplementary material for: Electrochemical imaging of thermochemical catalysis
Source: Nat Catal. 2026 Mar 5;9(3):307–18. doi: 10.1038/s41929-026-01486-y (PMC13031128; doi:10.1038/s41929-026-01486-y)
Supplement: Supplementary file 1 — Supplementary Figs. 1–19, Notes 1–3, Tables 1 and 2 and References. [file 41929_2026_1486_MOESM1_ESM.pdf]

nature catalysis

Supplementary information

https://doi.org/10.1038/s41929-026-01486-y

Electrochemical imaging of thermochemical catalysis

In the format provided by the authors and unedited

Table of Contents

Supplementary Figures 2
Supplementary Figure 1. Electron backscatter diffraction (EBSD) map of platinum (Pt).
Supplementary Figure 2. Electrochemical cleaning of Pt.
Supplementary Figure 3. Grain-dependent electrocatalytic FAOR(Ar).
Supplementary Figure 4. Atomic force microscopy (AFM) characterization of Pt.
Supplementary Figure 5. Surface area analysis.
Supplementary Figure 6. Schematic of SECCM pipette movement during the experiment.
Supplementary Figure 7. Grain-dependent electrocatalytic ORR.
Supplementary Figure 8. Predicted mixed potential and mixed current density.
Supplementary Figure 9. Grain-dependent electrocatalytic FAOR(O_2) and ORR(FA).
Supplementary Figure 10. EBSD grain orientation map of Pt.
Supplementary Figure 11. Grain-dependent electrocatalytic activity at 200 mV s-1.
Supplementary Figure 12. Grain-dependent mixed potentials and catalytic rates at 200 mV s-1.
Supplementary Figure 13. Schematic of a single-barrel SECCM pipette (not to scale), with meniscus contact with Pt substrate.
Supplementary Figure 14. Mixed potential comparison.
Supplementary Figure 15. Tafel slope.
Supplementary Figure 16. Thermocatalytic rate of FAOR.
Supplementary Figure 17. Catalytic rate comparison.
Supplementary Figure 18. Chemical cross-talk effect.
Supplementary Figure 19. Monitoring changes in crystal orientation of Pt.

Supplementary Notes 9
Supplementary Note 1: Grain-dependent reactions under a scan rate closer to steady-state conditions
Supplementary Note 2: The limiting current of FAOR under SECCM experimental conditions
Supplementary Note 3: Catalytic rate of thermochemical FAOR as current density for a bulk polycrystalline Pt

Supplementary Tables 17
Supplementary Table 1. Calculations of activity changes between FAOR(Ar) and ORR.
Supplementary Table 2. Calculations of chemical cross-talk effect.

Supplementary References 18

Supplementary Figures

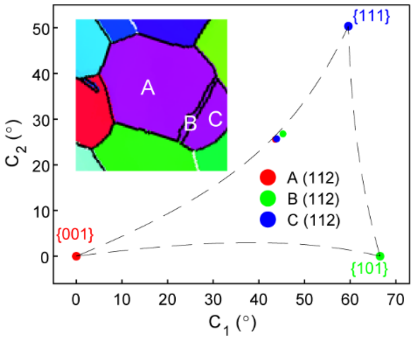

Supplementary Figure 1. Electron backscatter diffraction (EBSD) map of platinum (Pt). *Inverse pole figure of three independent grains (A, B, C) which are close to Pt(112), revealing small grain orientation variation. These grains were thus analyzed as one large Pt(112) grain. The figure displays the two-dimensional projection of crystallographic orientation relative to the low-index orientations in a face-centered cubic (fcc) crystal system, as reported earlier.*[1]

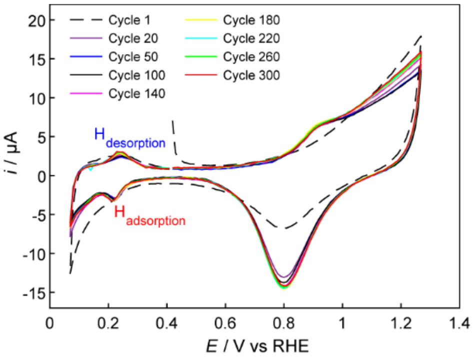

Supplementary Figure 2. Electrochemical cleaning of Pt. *A series of representative cyclic voltammograms (CVs) obtained from the electrochemical cleaning of the Pt electrode carried out before the scanning electrochemical cell microscopy (SECCM) experiments. The hydrogen adsorption ($H_{adsorption}$) and desorption ($H_{desorption}$) peaks are usually the main indicators for a clean Pt surface. Here, a standard three-electrode system was used for the cleaning, with a Pt coil as the counter electrode and a commercial leakless Ag/AgCl electrode (3.4 M KCl) as the reference electrode. Cyclic voltammetric measurements were performed on the Pt foil in deareated 0.1 M $HClO_4$.*

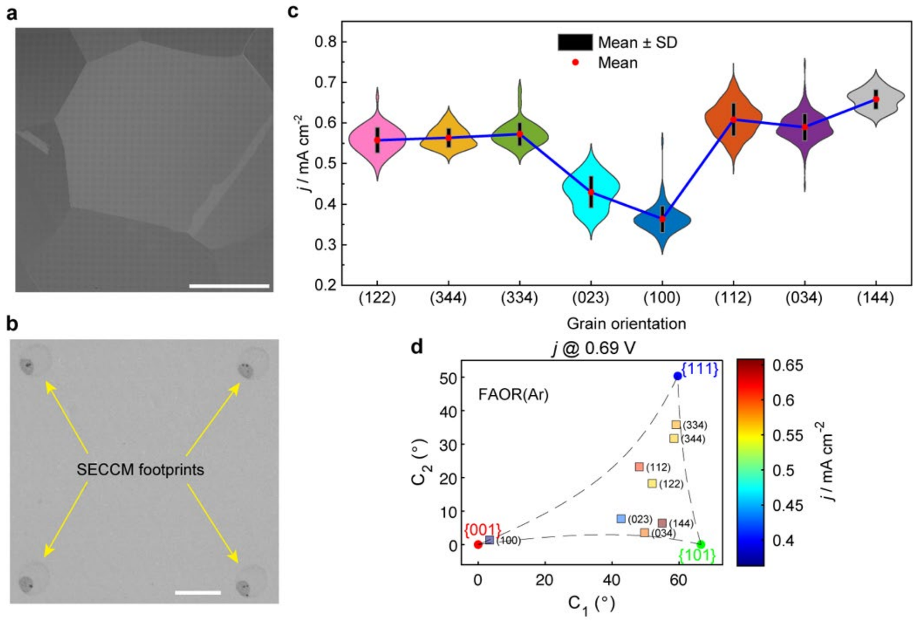

Supplementary Figure 3. Grain-dependent electrocatalytic FAOR(Ar). a) Scanning electron microscopy (SEM) image of the polycrystalline Pt surface after the SECCM FAOR(Ar) measurement, corresponding to Figure 2a. Scale bar is 100 µm. b) Magnified SEM image that shows four individual footprints left by the SECCM FAOR(Ar) measurement. Scale bar is 2 µm. c) Violin plot and d) inverse pole figure of current densities at 0.69 V for the FAOR(Ar) on the eight different Pt grains probed by SECCM. The error bars represent +/- standard deviation (SD) from the mean. The sample sizes for grains with orientation distributions close to the (122), (344), (334), (023), (100), (112), (034) and (144) planes are n = 78, 22, 43, 17, 94, 603, 199 and 44, respectively. The sample size for each orientation corresponds to the cumulative number of SECCM measurements on grains exhibiting that orientation.

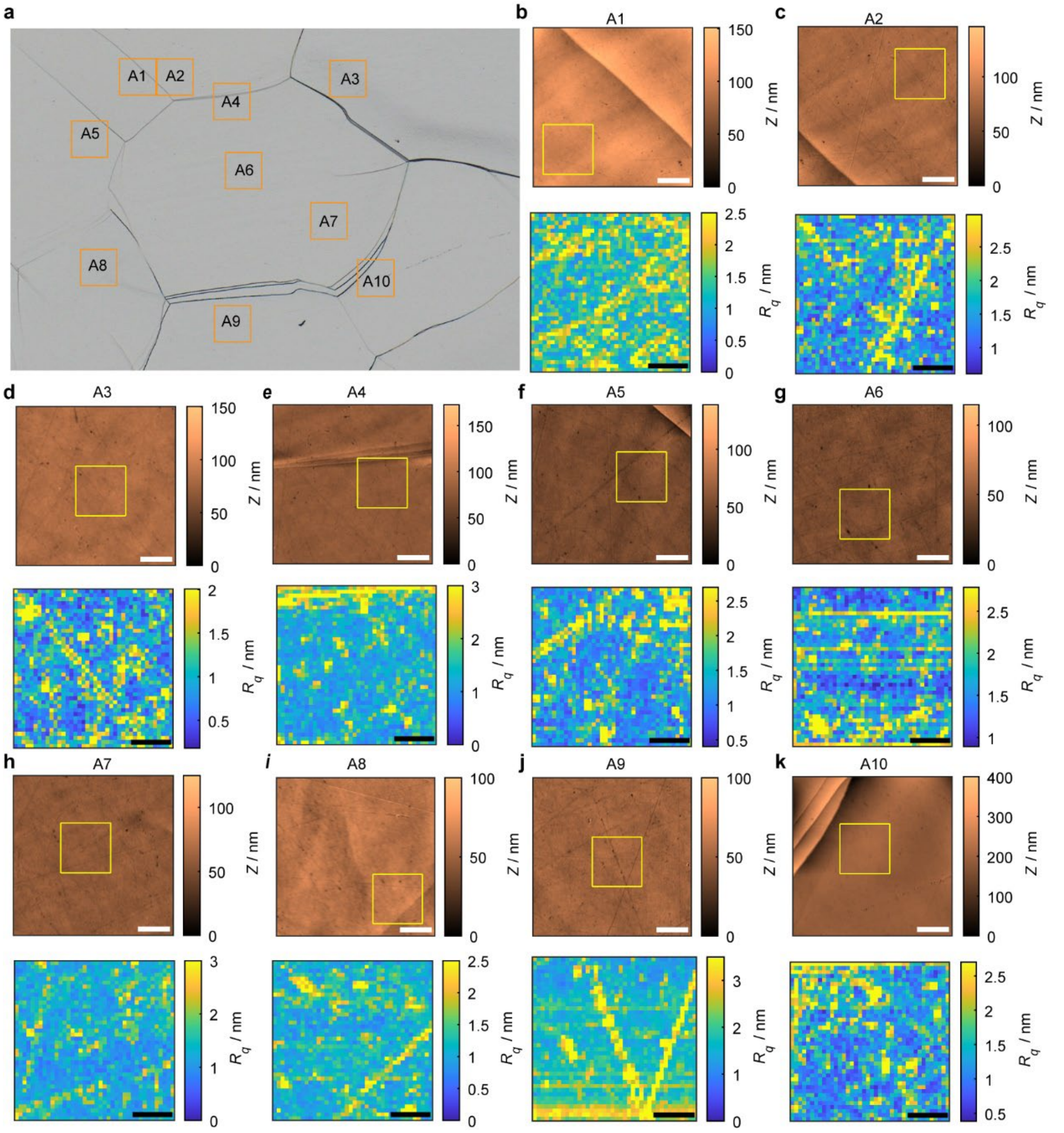

Supplementary Figure 4. Atomic force microscopy (AFM) characterization of Pt. *a) The optical image displays the polycrystalline Pt surface with ten selected areas (marked with orange squares and labeled from A1 to A10), each spanning various grains, for subsequent AFM measurements. The maps in panels b-k) depict the AFM topography (upper) and corresponding root mean square roughness (R_q, lower) of the selected areas marked with yellow squares from A1 to A10 on the Pt surface. The white and black scale bars are 10 and 4 µm, respectively.*

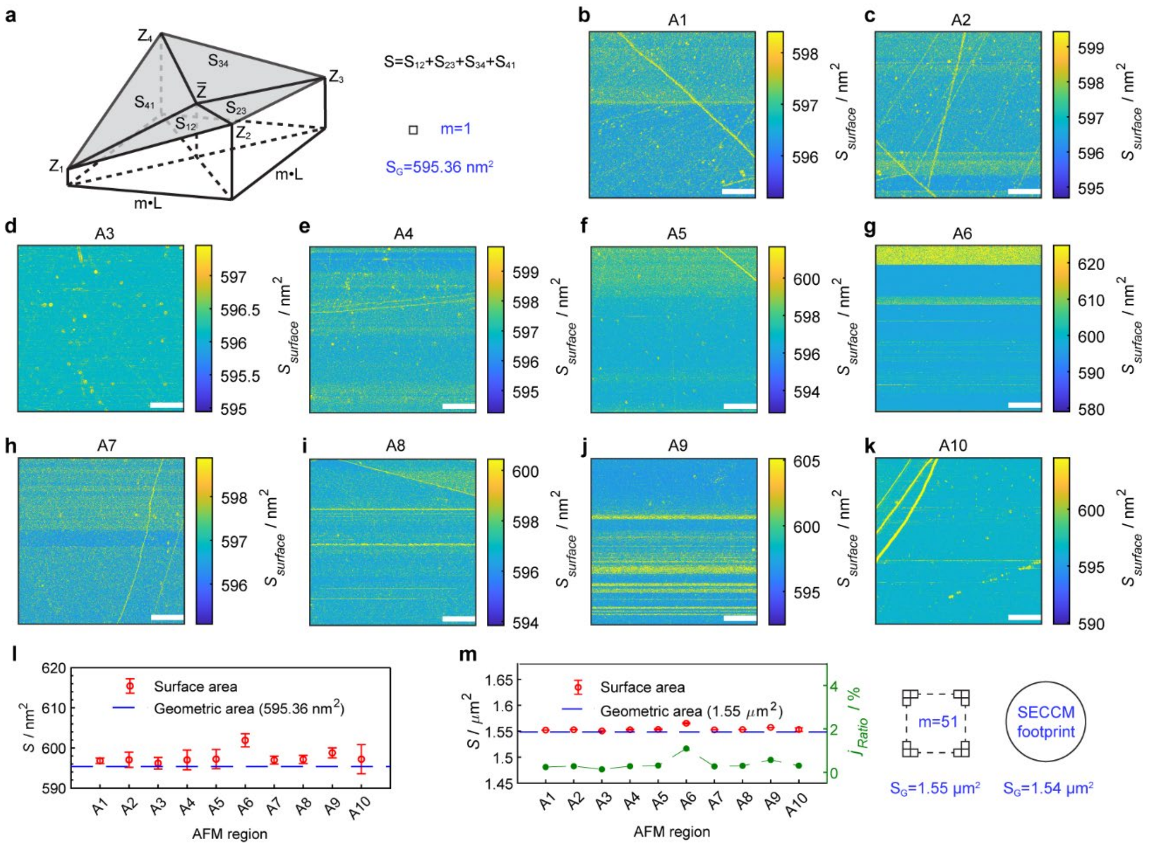

Supplementary Figure 5. Surface area analysis. *a) Surface area calculation triangulation scheme: The height values at four neighboring points (Z_1, Z_2, Z_3 and Z_4) define a local surface topology, with their mean height denoted as $\overline{Z}$. The surface area is estimated by summing the areas of four triangular subunits (S_{12}, S_{23}, S_{34} and S_{41}). The lateral resolution, L, corresponds to the pixel size in AFM mapping (Supplementary Figure 4b). When m=1, the projected area of a unit cell equals the geometric area S_G (595.36 nm^2), which serves as the reference for constructing surface area maps. The maps in panels b-k) depict the estimated surface area ($S_{surface}$) of the selected areas A1 to A10 on the Pt surface in Supplementary Figure 4a. The scale bars are 10 µm. l) The average surface area of the selected regions (A1-A10) is evaluated with the geometric area (blue line) as a reference, demonstrating the consistency between the estimated surface area and the geometric area. m) A total of 2601 unit cells were selected, with their combined geometric area matching the SECCM footprint. The summed surface area of these unit cells was averaged and compared with their total geometric area, confirming the consistency between surface and geometric areas. The green scatter plot illustrates the increasing ratio of current density when normalized by geometric rather than surface area, highlighting the negligible impact of using geometric area for normalization. The error bars represent the +/- SD from the mean, based on measurements from each different region. The sample size for Supplementary Figure 5l is n = 4190209. Each sample corresponds to one unit surface area, calculated on basis of Supplementary Figure 5a. The sample size for Supplementary Figure 5m is n = 1600. Each sample contains 2601 units of surface area, as illustrated in Supplementary Figure 5m.*

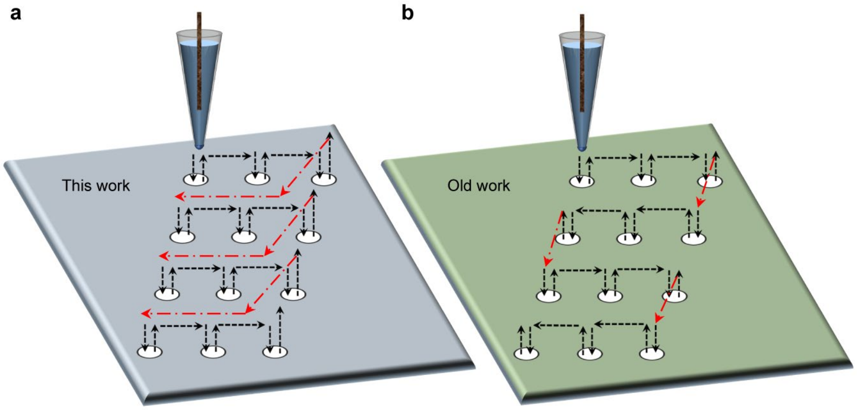

Supplementary Figure 6. Schematic of SECCM pipette movement during the experiment. *a) This work and (b) previous studies using old Warwick Electrochemical Scanning Probe Microscopy (WEC-SPM,* http://www.warwick.ac.uk/electrochemistry/wec-spm*) programs. The black arrow indicates pipette movement along the same line, while the red arrow represents repositioning at the end of each line.*

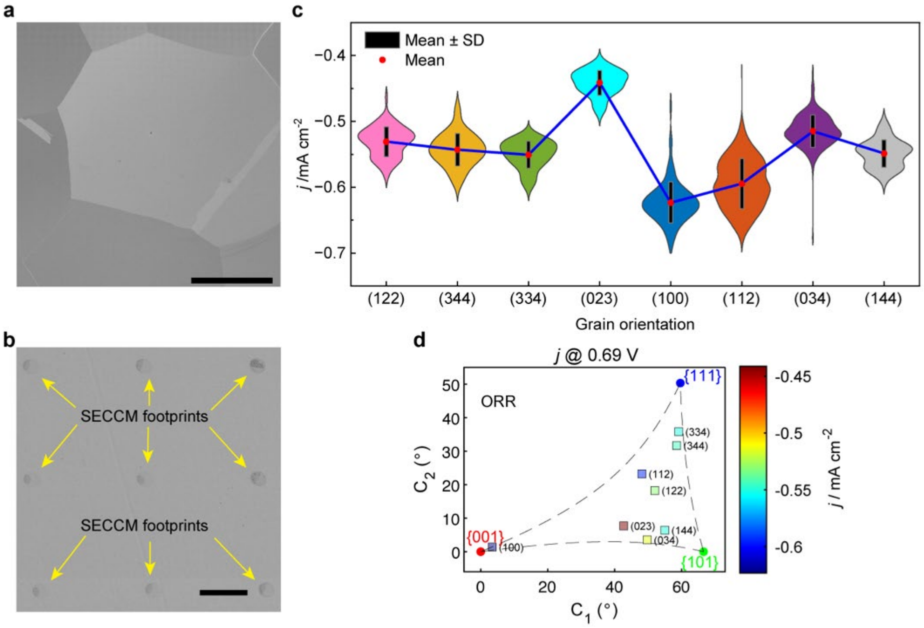

Supplementary Figure 7. Grain-dependent electrocatalytic ORR. *a) SEM image of the polycrystalline Pt surface after the SECCM ORR measurement, corresponding to Figure 2a. Scale bar is 100 µm. b) Magnified SEM image that shows nine individual footprints left by the SECCM ORR measurement. Scale bar is 4 µm. c) Violin plot and d) inverse pole figure of current densities at 0.69 V for the ORR on the eight different Pt grains probed by SECCM. The error bars represent +/- SD from the mean. The sample sizes for grains with orientation distributions close to the (122), (344), (334), (023), (100), (112), (034) and (144) planes are n = 78, 22, 43, 17, 94, 603, 199 and 44, respectively. The sample size for each orientation corresponds to the cumulative number of SECCM measurements on grains exhibiting that orientation.*

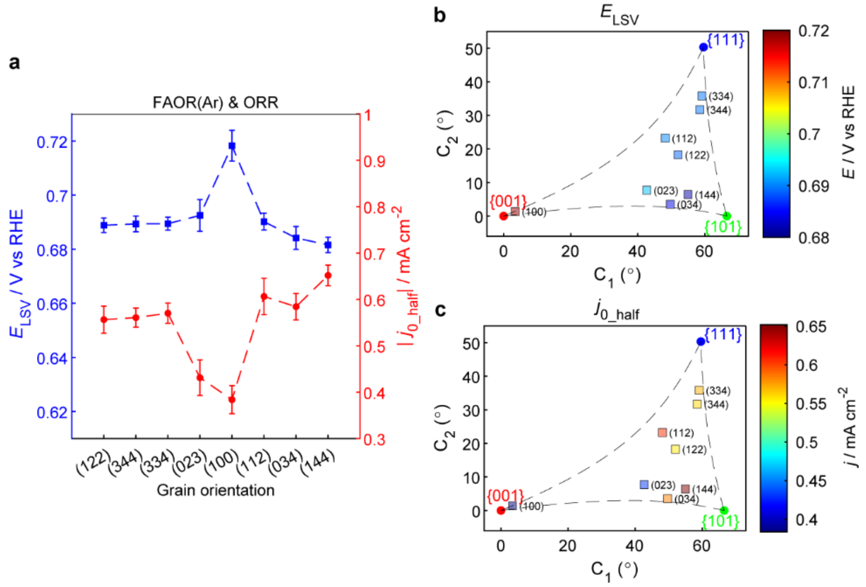

Supplementary Figure 8. Predicted mixed potential and mixed current density. *a) Grain-average current equivalency potential (E_{LSV}) and mixed current (j_{0_half}) obtained from the mixed potential theory comparing the voltammetric curves of the two isolated half-reactions, FAOR(Ar) and ORR. Inverse pole figures of b) E_{LSV} and c) j_{0_half} at current equivalency point as a function of Pt grain orientation. The error bars represent +/- SD from the mean. The sample sizes for grains with orientation distributions close to the (122), (344), (334), (023), (100), (112), (034) and (144) planes are n = 78, 22, 43, 17, 94, 603, 199 and 44, respectively. The sample size for each orientation corresponds to the cumulative number of SECCM measurements on grains exhibiting that orientation.*

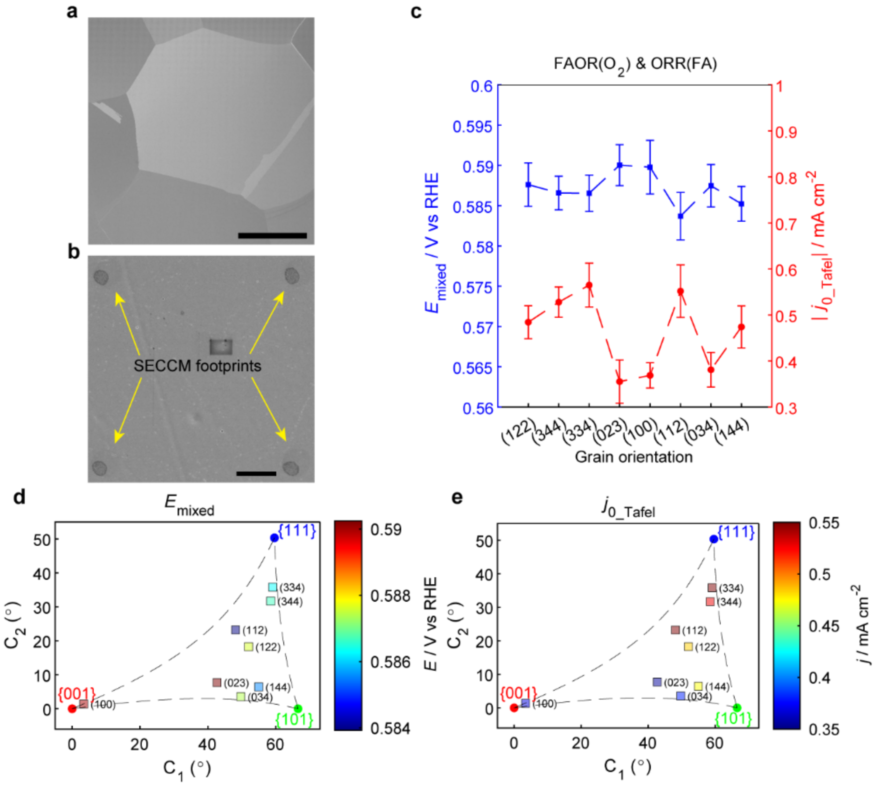

Supplementary Figure 9. Grain-dependent electrocatalytic FAOR(O_2) and ORR(FA). *a) SEM image of the polycrystalline Pt surface after the SECCM FAOR(O_2) measurement, corresponding to Figure 2a. Scale bar is 100 µm. b) Magnified SEM image that shows four individual footprints left by the SECCM FAOR(O_2) measurement. Scale bar is 2 µm. c) Grain-average zero current potential (E_{mixed}) and mixed current (j_{0_Tafel}) obtained from the mixed reactions in presence of co-reactant, FAOR(O_2). Inverse pole figures of d) E_{mixed} and e) j_{0_Tafel} at E_{Tafel} as a function of Pt grain orientation. The error bars represent +/- SD from the mean. The sample sizes for grains with orientation distributions close to the (122), (344), (334), (023), (100), (112), (034) and (144) planes are n = 86, 24, 42, 17, 95, 650, 212 and 47, respectively. The sample size for each orientation corresponds to the cumulative number of SECCM measurements on grains exhibiting that orientation.*

Supplementary Notes

Supplementary Note 1: Grain-dependent reactions under a scan rate closer to steady-state conditions

Achieving a true steady-state is challenging under the fast scan rate used. However, SECCM benefits from conical diffusion at the nano-pipette, allowing higher scan rates for kinetic analysis compared to planar diffusion-limited macroscale experiments. The fast scan rate was chosen for efficient mapping of grain-dependent activity across a large area. To address this concern, we conducted an additional SECCM experiment at a slower scan rate (200 mV s^{-1}). While this reduced lateral resolution, the grain-dependent activity persisted, suggesting that the structure-dependent activity we report is relevant under conditions closer to steady-state.

Another Pt surface, containing four distinct grains within the probed region (Supplementary Figure 10) with orientations close to (314), (344), (334) and (114) planes, was analyzed. The spatially resolved equipotential frame at 0.73 V (Supplementary Figure 11a, derived from Supplementary Video 4), corresponding to the expected zero net current potential on average, reveals grain-dependent FAOR(Ar) activity. Similarly, the equipotential map at 0.73 V (Supplementary Figure 11b, derived from Supplementary Video 5) displays heterogeneous, grain-dependent ORR activity.

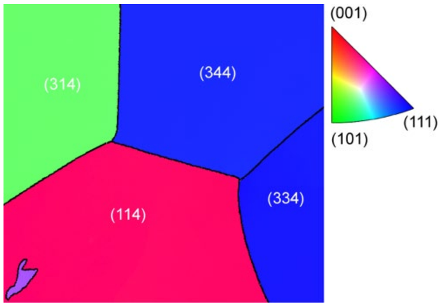

Supplementary Figure 10. EBSD grain orientation map of Pt. *The second polycrystalline Pt surface probed during the SECCM experiments at a voltammetric scan rate of 200 mV s^{-1}.*

Moreover, spatially resolved maps for ORR(FA) at 0.60 V (Supplementary Figure 11c, derived from Supplementary Video 6) and FAOR(O_2) at 0.80 V (Supplementary Figure 11d, derived from Supplementary Video 6) further confirm strong grain-dependent variations when both reactants are present. Results obtained at a slower scan rate (200 mV s^{-1}) exhibit consistent grain dependency compared to those collected at 1 V s^{-1}. Notably, grain dependency is also evident in E_{LSV} (Supplementary Figure 12a-12b) and E_{mixed} (Supplementary Figure 12c-12d) at 200 mV s^{-1}, though both values shift positively relative to 1 V s^{-1}.

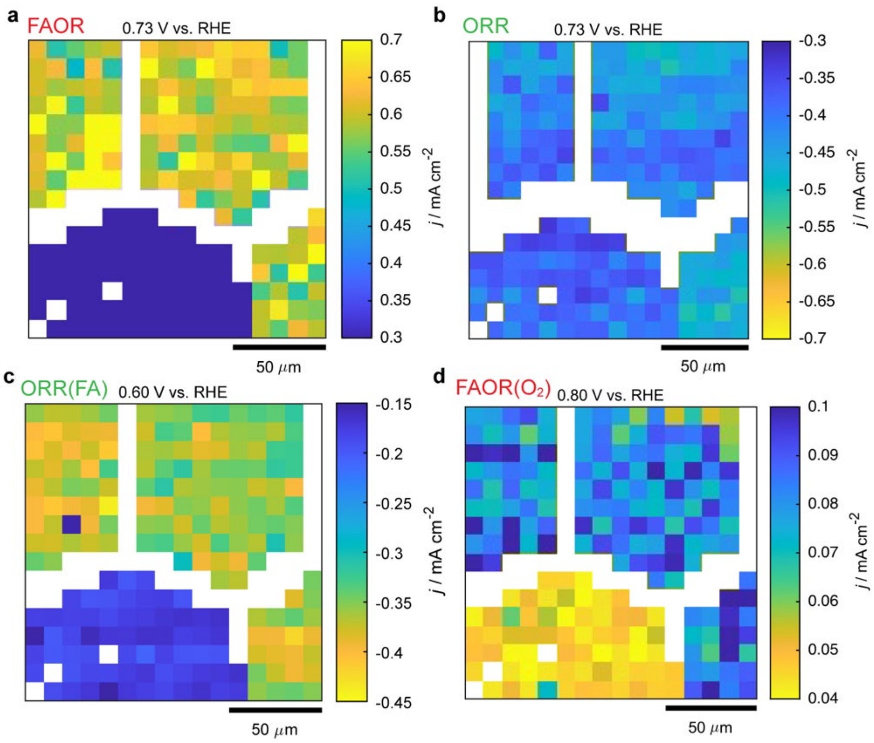

Supplementary Figure 11. Grain-dependent electrocatalytic activity at 200 mV s^{-1}. *a) Spatially-resolved equipotential snapshot of FAOR(Ar) obtained by SECCM (Supplementary Video 4) at a potential of 0.73 V using 0.5 M FA in 0.1 M $HClO_4$ under a continuous Ar flow. b) Spatially-resolved equipotential snapshot of ORR obtained by SECCM (Supplementary Video 5) at a potential of 0.73 V using 0.1 M $HClO_4$ under a continuous O_2 flow. Spatially-resolved equipotential snapshots of FAOR under a continuous O_2 flow obtained by SECCM (Supplementary Video 6) at potentials of c) 0.60 and d) 0.80 V, respectively.*

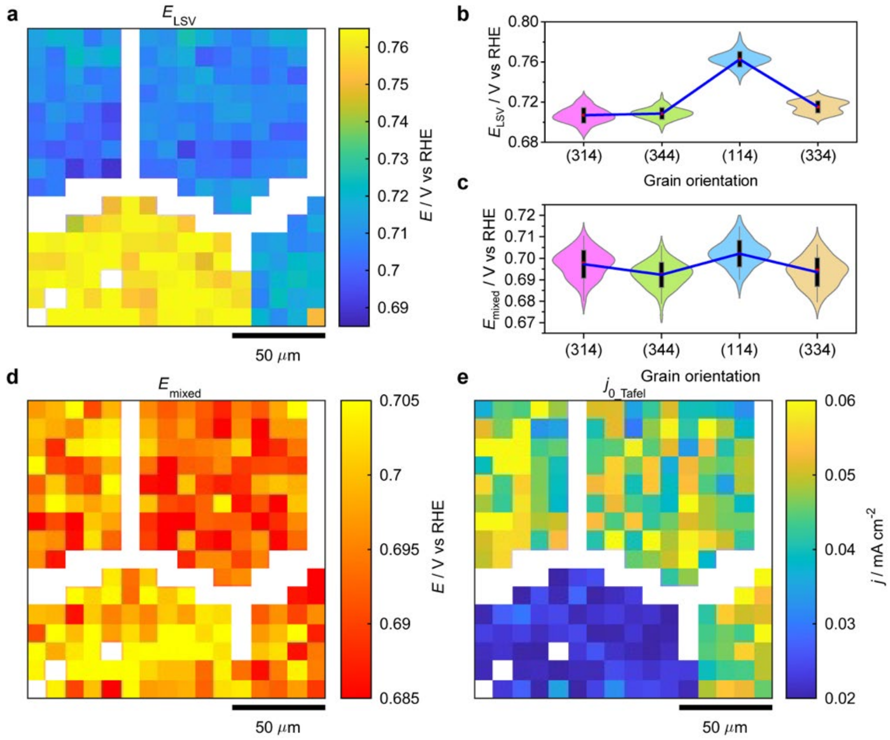

Supplementary Figure 12. Grain-dependent mixed potentials and catalytic rates at 200 mV s^{-1}. *a) E_{LSV} map produced from all SECCM spots, based on the mentioned calculation in Figure 3a. b) Violin plot of E_{LSV} on the four different Pt grains probed during the SECCM experiments. c) Violin plot of E_{mixed} for the four different Pt grains probed by SECCM. d) Spatially-resolved mixed potential (E_{mixed}) map, built using the potential value where the net current equals zero. e) SECCM spatially-resolved map of j_{0_Tafel} calculated from the Tafel analysis. Note that some pixels in these maps where left blank white to account for a small lateral shift between corresponding pixels for the two independent SECCM experiments, particularly to avoid mismatch in pixels close to grain boundaries. The error bars represent +/- SD from the mean. The sample sizes of E_{LSV} for grains with orientation distributions close to the (314), (344), (114) and (334) planes are n = 42, 79, 65 and 23, respectively. The sample sizes of E_{mixed} for grains with orientation distributions close to the (314), (344), (114) and (334) planes are n = 41, 79, 64 and 23, respectively. The sample size for each orientation corresponds to the cumulative number of SECCM measurements on grains exhibiting that orientation.*

Supplementary Note 2: The limiting current of FAOR under SECCM experimental conditions

The SECCM limiting current under experimental conditions was calculated using two established methods (Method 1[2] and Method 2[3,4]), which yield consistent results depending on the estimation approach.

Method 1:

As illustrated in Supplementary Figure 13, r_p represents the SECCM pipette radius, γ is the pipette half-angle (typically ~5°–15°), and h denotes the meniscus height. The number of electrons transferred is given by n, while D[5] and c correspond to the diffusion coefficient and concentration of formate, respectively. Additionally, R represents the equivalent electrode radius, and S denotes the geometric area of the SECCM footprint.

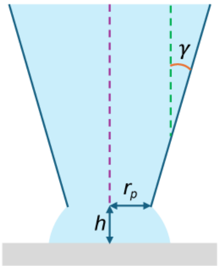

Supplementary Figure 13. Schematic of a single-barrel SECCM pipette (not to scale), with meniscus contact with Pt substrate. *The r_p represents the SECCM pipette radius, γ is the pipette half-angle (typically ~5°–15°), and h denotes the meniscus height.*

When γ=5°,

$$R_{\gamma=5^\circ} = \frac{r_p}{\sin\gamma} - \frac{h}{\cos\gamma} = \frac{500}{\sin 5^\circ} - \frac{250}{\cos 5^\circ} = 5.49\times10^3\ nm \qquad (1)$$

$$\begin{aligned} i_{\lim(\gamma=5^\circ)} &= 2\pi R_{\gamma=5^\circ} nFDc = 2\pi\times5.49\times10^3\ nm\times2\times9.6485\times10^4\ C\cdot mol^{-1}\times1.454\times10^{-5}\ cm^2\cdot s^{-1}\times0.5\ mol\cdot L^{-1} \\ &= 2\pi\times5.49\times10^3\ nm\times2\times9.6485\times10^4\times A\cdot s\times mol^{-1}\times1.454\times10^{-5}(10^7\ nm)^2\cdot s^{-1}\times0.5\times mol\cdot((10^8\ nm)^3)^{-1} \qquad (2) \\ &= 4.839\times10^{-3}\ mA \end{aligned}$$

$$j^{SECCM}_{\lim(\gamma=5^\circ)} = i_{\lim(\gamma=5^\circ)}(1-\cos\gamma)/S = \frac{4.839\times10^{-3}\times(1-\cos 5^\circ)}{1.54\times10^{-8}} = 1200\ mA\cdot cm^{-2} \qquad (3)$$

When γ=15°,

$$R_{\gamma=15^\circ} = \frac{r_p}{\sin\gamma} - \frac{h}{\cos\gamma} = \frac{500}{\sin 15^\circ} - \frac{250}{\cos 15^\circ} = 1.67\times10^3\ nm \qquad (4)$$

$$\begin{aligned} i_{\lim(\gamma=15^\circ)} &= 2\pi R_{\gamma=15^\circ} nFDc = 2\pi\times1.67\times10^3\ nm\times2\times9.6485\times10^4\ C\cdot mol^{-1}\times1.454\times10^{-5}\ cm^2\cdot s^{-1}\times0.5\ mol\cdot L^{-1} \\ &= 2\pi\times1.67\times10^3\ nm\times2\times9.6485\times10^4\times A\cdot s\times mol^{-1}\times1.454\times10^{-5}(10^7\ nm)^2\cdot s^{-1}\times0.5\times mol\cdot((10^8\ nm)^3)^{-1} \qquad (5) \\ &= 1.472\times10^{-3}\ mA \end{aligned}$$

$$j^{SECCM}_{\lim(\gamma=15^\circ)} = i_{\lim(\gamma=15^\circ)}(1-\cos\gamma)/S = \frac{1.472\times10^{-3}\times(1-\cos 15^\circ)}{1.54\times10^{-8}} = 3257\ mA\cdot cm^{-2} \qquad (6)$$

Method 2:

Generally, 1/10th of the limiting current of a disk electrode is used to estimate the current in SECCM experiments.

$$
\begin{aligned}
i_{\lim} &\approx 4nr_pFDc/10 = 4\times 2\times 500\,nm\times 9.6485\times 10^4\ C\cdot mol^{-1}\times 1.454\times 10^{-5}\ cm^2\cdot s^{-1}\times 0.5\,mol\cdot L^{-1}/10 \\
&= 4\times 2\times 500\,nm\times 9.6485\times 10^4\times A\cdot s\times mol^{-1}\times 1.454\times 10^{-5}(10^7\ nm)^2\cdot s^{-1}\times 0.5\times mol\cdot((10^8\ nm)^3)^{-1}/10 \quad (7) \\
&= 2.806\times 10^{-5}\ mA
\end{aligned}
$$

$$
j_{\lim}^{SECCM} = i_{\lim}/S = \frac{2.806\times 10^{-5}}{1.54\times 10^{-8}} = 1822\,mA\cdot cm^{-2} \qquad (8)
$$

Based on our estimations, the calculated current density is 2-3 orders of magnitude higher than the observed values, indicating that under our reaction conditions, the process is not mass transport-limited. Instead, CO and adsorbed formate likely poison Pt active sites, as has been previously observed[6], leading to the observed current plateau.

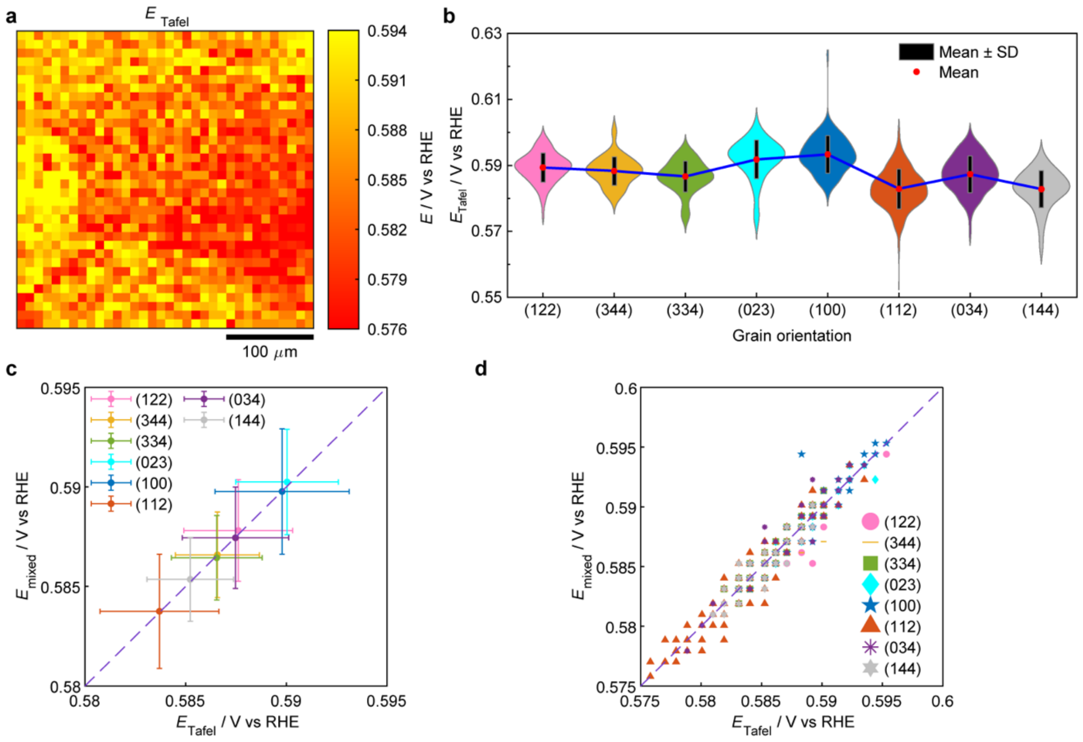

Supplementary Figure 14. Mixed potential comparison. *a) Spatially-resolved E_{Tafel} map, obtained from Tafel analysis. b) Violin plot of E_{Tafel} for the eight different Pt grains from Tafel analysis. c) Relationship between the average E_{mixed} and the average E_{Tafel} on the eight different Pt grains probed with SECCM. d) Relationship between E_{mixed} and E_{Tafel} of all SECCM spots. Dashed line indicates perfect correspondence. The error bars represent the standard deviation from the mean, based on measurements from each different grain. The error bars of E_{mixed} and E_{Tafel} represent +/- SD from the mean. The sample sizes for grains with orientation distributions close to the (122), (344), (334), (023), (100), (112), (034) and (144) planes are n = 86, 24, 42, 17, 95, 650, 212 and 47, respectively. The sample size for each orientation corresponds to the cumulative number of SECCM measurements on grains exhibiting that orientation.*

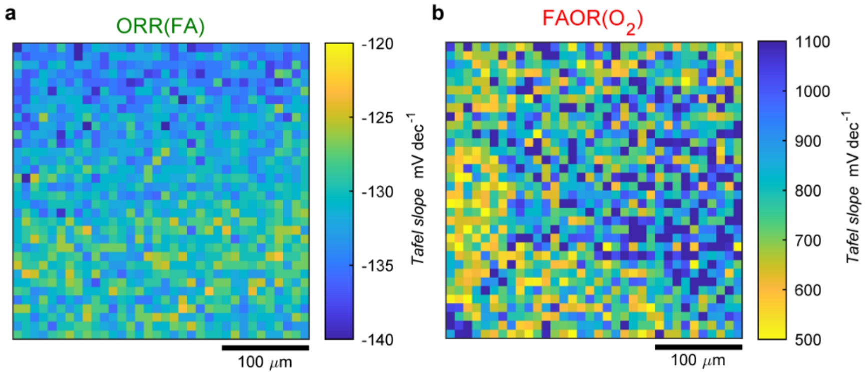

Supplementary Figure 15. Tafel slope. *a) Spatially-resolved Tafel slope map for ORR(FA) dominated process in 0.44-0.54 V potential range, obtained from Tafel analysis, in line with macroscale measurements for ORR on Pt[7,8]. b) Spatially-resolved Tafel slope map for FAOR(O_2) dominated process in 0.66-0.76 V potential range, obtained from Tafel analysis, in agreement with our previous results observed on carbon-supported Pt nanoparticles[9].*

Supplementary Note 3: Catalytic rate of thermochemical FAOR as current density for a bulk polycrystalline Pt

A Pt foil (Thermo Fisher Scientific, 99.9%, 0.025mm thick) of 10.5 cm^2 area was used as the catalyst. The foil was annealed under a butane flame and then quickly quenched in deionized water 3 times, analogously to the Pt foil preparation used for SECCM analysis. The Pt foil was then electrochemically cleaned by cyclic voltammetry in a standard three-electrode system, using a Pt mesh as the counter electrode and a commercial leakless Ag/AgCl electrode (eDAQ) as the reference electrode. Cyclic voltammetry was performed on the Pt foil in dearated 0.1M $HClO_4$, scanning from 0 to 1 V vs RHE at 100 mV s^{-1} for 10 cycles.

The ensemble rate of aerobic formic acid oxidation on this electrode was measured using gas chromatography. The Pt foil electrode was placed in a 15 mL solution of 0.1 M $HClO_4$ with 0.5 M FA, saturated with O_2 at 1 atm. An O_2 stream was bubbled through the cell at 10 sccm, and the outlet was sent to a gas chromatograph (GC, SRI Instruments, multi-gas analyzer 3) to quantify the amount of CO_2 formed. The steady-state rate was measured over 60 minutes, during which five GC injections were taken. The rate of CO_2 production was averaged across five data points to monitor the reaction (Supplementary Figure 16). The equivalent current density (j_{CO_2}) for aerobic FAOR on the Pt foil was calculated from the concentration of CO_2 in the gas stream leaving the cell using the following equation:

$$j_{CO_2} = cnFV \times \frac{P}{RT} \times \frac{1}{A} \qquad (9)$$

Where c is the CO_2 mole fraction in the gas stream (measured by GC), n is the number of electrons transferred per mole of CO_2 produced (2 e^- for CO_2 from FA, $HCOOH \rightarrow CO_2 + 2H^+ + 2e^-$), F is the Faraday constant, V is the volumetric flow rate of the gas stream, P is the pressure of the gas stream (1 atm), R is the gas constant, T is the gas temperature (298 K), and A is the surface area of the Pt foil.

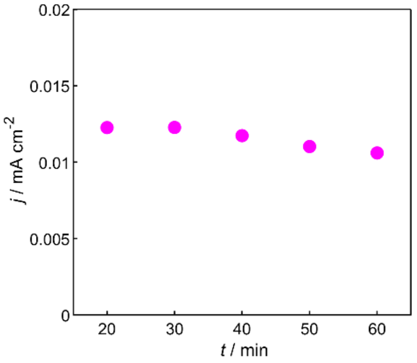

Supplementary Figure 16. Thermocatalytic rate of FAOR. *Steady-state rates of aerobic FA oxidation measured on a Pt foil in a 0.1 M* $HClO_4$ *solution containing 0.5 M FA, under 1 atm of* O_2*. During catalysis, the open circuit potential ranged from 0.48 V to 0.47 V vs RHE. The average oxidation rate was 0.012 ± 0.001 mA* cm^{-2}*. The solution was sparged with* O_2 *for 20 minutes before the first GC injection.*

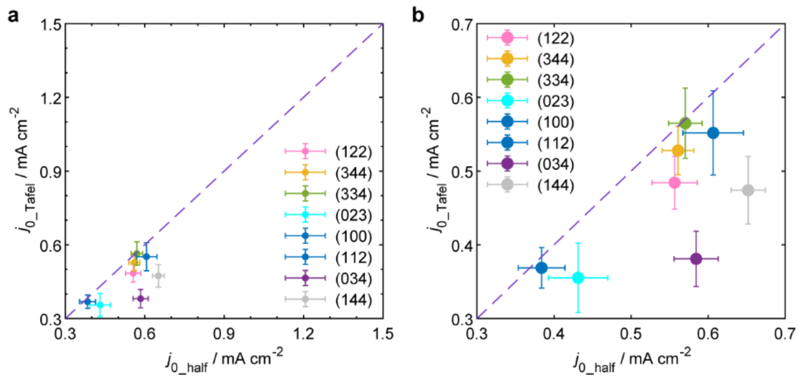

Supplementary Figure 17. Catalytic rate comparison. *a) Relationship between the mixed current density obtained from individual half reactions (*j_{0_half}*) and Tafel analysis (*j_{0_Tafel}*) on the eight different Pt grains probed with SECCM. Dashed line indicates perfect correspondence. b) Magnified version of the plot in (a). The error bars represent the standard deviation from the mean, based on measurements from each different grain. The error bars represent +/- SD from the mean of both* j_{0_Tafel} *and* j_{0_half}*. The sample sizes of* j_{0_half} *for grains with orientation distributions close to the (122), (344), (334), (023), (100), (112), (034) and (144) planes are n = 78, 22, 43, 17, 94, 603, 199 and 44, respectively. The sample sizes of* j_{0_Tafel} *for grains with orientation distributions close to the (122), (344), (334), (023), (100), (112), (034) and (144) planes are n = 86, 24, 42, 17, 95, 650, 212 and 47, respectively. The sample size for each orientation corresponds to the cumulative number of SECCM measurements on grains exhibiting that orientation.*

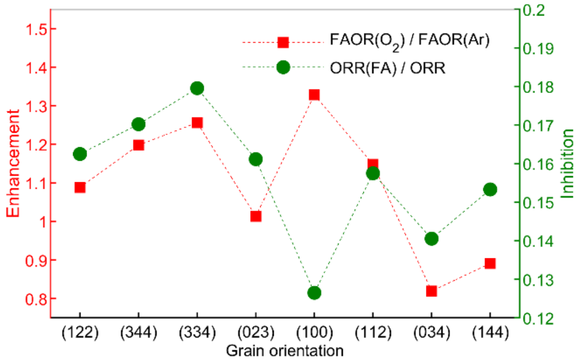

Supplementary Figure 18. Chemical cross-talk effect. *The ratio between average mixed current densities (j_{0_Tafel}) obtained at mixed potential (E_{mixed}) for the mixed reactions, and the average current densities extracted at E_{mixed} for the half-reactions, i.e. ORR(FA)/ORR (green marks) and FAOR(O_2)/FAOR(Ar) (red marks). This calculation represents the grain-dependent inhibition effect of FA on the ORR and enhancement effect of O_2 on the FAOR.*

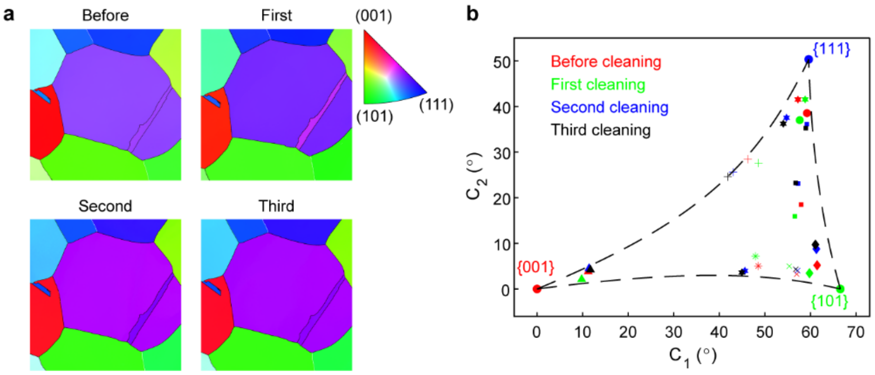

Supplementary Figure 19. Monitoring changes in crystal orientation of Pt. *a) EBSD maps of Pt surface before any electrochemical cleaning process and after one, two and three cycles of UV and electrochemical cleaning. b) Inverse pole figures showing the grain orientation changes for the pristine and cleaned surface, which reveal only small changes in the surface orientation after cleaning.*

Supplementary Tables

Supplementary Table 1. Calculations of activity changes between FAOR(Ar) and ORR. *Average current densities and standard deviations obtained for FAOR(Ar) and ORR at 0.69 V as a function of grain orientation, also indicating the relative change in average current density between FAOR(Ar) and ORR.*

Grain orientation	*FAOR(Ar) $mA \cdot cm^{-2}$*		*ORR $mA \cdot cm^{-2}$*		*$(Mean_{\_FAOR(Ar)} - \lvert Mean_{\_ORR} \rvert) / \lvert Mean_{\_ORR} \rvert$ %*
	$Mean_{\ FAOR(Ar)}$	*$SD_{\ FAOR(Ar)}$*	*$Mean_{\ ORR}$*	*$SD_{\ ORR}$*	
(122)	0.56	0.03	-0.53	0.02	5.05
(344)	0.56	0.02	-0.54	0.02	3.70
(334)	0.57	0.03	-0.55	0.02	3.94
(023)	0.43	0.04	-0.44	0.02	-2.63
(100)	0.36	0.03	-0.62	0.03	-41.69
(112)	0.61	0.04	-0.59	0.04	2.29
(034)	0.59	0.03	-0.51	0.02	14.53
(144)	0.66	0.02	-0.55	0.02	19.88

Supplementary Table 2. Calculations of chemical cross-talk effect. *A series of current density and mixed current density values for FAOR(Ar), ORR, $FAOR(O_2)$ and ORR(FA) at E_{mixed} as a function of grain orientation.*

Grain orientation		(122)	(344)	(334)	(023)	(100)	(112)	(034)	(144)
FAOR(Ar)	j_a @ E_{mixed} / $mA \cdot cm^{-2}$	0.45	0.44	0.45	0.35	0.28	0.48	0.46	0.53
$FAOR(O_2)$	$j_{0\ Tafel}$ / $mA \cdot cm^{-2}$	0.48	0.53	0.56	0.36	0.37	0.55	0.38	0.47
$j_{0\ Tafel} - j_a$ / $mA \cdot cm^{-2}$		0.03	0.09	0.11	0.01	0.09	0.07	-0.08	-0.06
$\frac{j_{0\_Tafel}}{j_a}$		1.07	1.20	1.24	1.03	1.32	1.15	0.83	0.89
ORR	$\lvert j_c \rvert$ @ E_{mixed} / $mA \cdot cm^{-2}$	2.98	3.1	3.15	2.2	2.92	3.5	2.71	3.09
ORR(FA)	$\lvert j_{0\ Tafel} \rvert$ / $mA \cdot cm^{-2}$	0.48	0.53	0.56	0.36	0.37	0.55	0.38	0.47
$\lvert j_{0\ Tafel} - j_c \rvert$ / $mA \cdot cm^{-2}$		2.50	2.57	2.59	1.84	2.55	2.95	2.33	2.62
$\lvert \frac{j_{0\_Tafel}}{j_c} \rvert$		0.16	0.17	0.18	0.16	0.13	0.16	0.14	0.15

Supplementary References

1. Daviddi, E. *et al.* Nanoscale electrochemistry in a copper/aqueous/oil three-phase system: surface structure-activity-corrosion potential relationships. *Chem. Sci.* **12**, 3055-3069 (2020).
2. Anderson, K. L. & Edwards, M. A. Evaluating Analytical Expressions for Scanning Electrochemical Cell Microscopy (SECCM). *Anal. Chem.* **95**, 8258-8266 (2023).
3. Snowden, M. E. *et al.* Scanning electrochemical cell microscopy: theory and experiment for quantitative high resolution spatially-resolved voltammetry and simultaneous ion-conductance measurements. *Anal. Chem.* **84**, 2483-2491 (2012).
4. Bentley, C. L. Scanning electrochemical cell microscopy for the study of (nano)particle electrochemistry: From the sub-particle to ensemble level. *Electrochem Sci Adv.* **2** (2021).
5. Lide, D. R. *CRC Handbook of Chemistry and Physics*. 85th ed, (CRC Press, 2005).
6. Schwarz, K. A., Sundararaman, R., Moffat, T. P. & Allison, T. C. Formic acid oxidation on platinum: a simple mechanistic study. *Phys. Chem. Chem. Phys.* **17**, 20805-20813 (2015).
7. Gómez-Marín, A. M., Rizo, R. & Feliu, J. M. Oxygen reduction reaction at Pt single crystals: a critical overview. *Catal. Sci. Technol.* **4**, 1685-1698 (2014).
8. Wang, J. X., Markovic, N. M. & Adzic, R. R. Kinetic Analysis of Oxygen Reduction on Pt(111) in Acid Solutions: Intrinsic Kinetic Parameters and Anion Adsorption Effects. *J. Phys. Chem. B* **108**, 4127-4133 (2004).
9. Ryu, J. *et al.* Thermochemical aerobic oxidation catalysis in water can be analysed as two coupled electrochemical half-reactions. *Nat. Catal.* **4**, 742-752 (2021).
